# Assessment of New Coronary Features on Quantitative Coronary Angiographic Images With Innovative Unsupervised Artificial Adaptive Systems: A Proof-of-Concept Study

**DOI:** 10.3389/fcvm.2021.730626

**Published:** 2021-10-14

**Authors:** Mauro Amato, Massimo Buscema, Giulia Massini, Guido Maurelli, Enzo Grossi, Beatrice Frigerio, Alessio L. Ravani, Daniela Sansaro, Daniela Coggi, Cristina Ferrari, Antonio L. Bartorelli, Fabrizio Veglia, Elena Tremoli, Damiano Baldassarre

**Affiliations:** ^1^Centro Cardiologico Monzino, Istituto di Ricovero e Cura a Carattere Scientifico (IRCCS), Milan, Italy; ^2^Semeion, Research Centre of Sciences of Communication, Rome, Italy; ^3^Department of Mathematical and Statistical Sciences, University of Colorado Denver, Denver, CO, United States; ^4^Department of Biomedical and Clinical Sciences “Luigi Sacco”, University of Milan, Milan, Italy; ^5^Maria Cecilia Hospital, Cotignola, Italy; ^6^Department of Medical Biotechnology and Translational Medicine, Università degli Studi di Milano, Milan, Italy

**Keywords:** atherosclerosis, diagnostic, coronary angiography, artificial intelligence, ultrasonography, image processing, computer-assisted

## Abstract

**Background and Purpose:** The Active Connection Matrixes (ACMs) are unsupervised artificial adaptive systems able to extract from digital images features of interest (edges, tissue differentiation, etc.) unnoticeable with conventional systems. In this proof-of-concept study, we assessed the potentiality of ACMs to increase measurement precision of morphological structures (e.g., stenosis and lumen diameter) and to grasp morphological features (arterial walls) from quantitative coronary angiography (QCA), unnoticeable on the original images.

**Methods:** Archive images of QCA and intravascular ultrasound (IVUS) of 10 patients (8 men, age 69.1 ± 9.7 years) who underwent both procedures for clinical reasons were retrospectively analyzed. Arterial features derived from “IVUS images,” “conventional QCA images,” and “ACM-reprocessed QCA images” were measured in 21 coronary segments. Portions of 1-mm length (263 for lumen and 526 for arterial walls) were head-to-head compared to assess quali-quantitative between-methods agreement.

**Results:** When stenosis was calculated on “ACM-reprocessed QCA images,” the bias vs. IVUS (gold standard) did not improve, but the correlation coefficient of the QCA–IVUS relationship increased from 0.47 to 0.83. When IVUS-derived lumen diameters were compared with diameters obtained on ACM-reprocessed QCA images, the bias (−0.25 mm) was significantly smaller (*p* < 0.01) than that observed with original QCA images (0.58 mm). ACMs were also able to extract arterial wall features from QCA. The bias between the measures of arterial walls obtained with IVUS and ACMs, although significant (*p* < 0.01), was small [0.09 mm, 95% CI (0.03, 0.14)] and the correlation was fairly good (*r* = 0.63; *p* < 0.0001).

**Conclusions:** This study provides proof of concept that ACMs increase the measurement precision of coronary lumen diameter and allow extracting from QCA images hidden features that mirror well the arterial walls derived by IVUS.

## Introduction

Historically, analysis of luminal stenosis by quantitative coronary angiography (QCA) has been the reference method for angiographic diagnosis of coronary narrowing (1, 2), for guiding revascularization procedures (1), and for the assessment of coronary artery disease (CAD) (3). QCA, however, provides only two-dimensional images of luminal narrowing and does not provide any direct information on the arterial wall. Moreover, QCA may underestimate stenosis severity in patients with low-grade and/or eccentric atherosclerotic lesions (4, 5).

Nowadays, diagnosis and therapeutic decision-making processes about coronary atheroma are supported by the intravascular ultrasound (IVUS), which allows the direct assessment of coronary arterial wall geometry (6). Unfortunately, the invasive nature of IVUS limits its use in coronary characterization. Furthermore, IVUS can only explore vessels large enough to allow the unrestricted passage of the ultrasound transducer (7). Therefore, despite being widely used by interventional cardiologists, IVUS does not fully solve the problem of the lack of information on coronary arterial walls.

Experimental evidences show that diagnostic images produced by conventional imaging techniques contain more information than usually believed (8–10). Such information, however, is often not visible even to skilled observers, since they emerge from between-pixels relationships/interactions too complex to be grasped by conventional mathematical systems. The Active Connection Matrixes (ACMs) are a family of unsupervised artificial adaptive systems able to automatically extract features of interest (e.g., edges and tissue differentiation) from digital images when activated by original non-linear equations. ACM activation allows the reduction of image noise while maintaining the spatial resolution of high contrast structures and the expression of hidden morphological features (11). This proof-of-concept study aimed at investigating the potentiality of ACMs to grasp additional information from QCA images.

## Materials and Methods

### Coronary Segments Analyzed

This retrospective study, performed by using archive images collected between 2012 and 2013, was approved by the local Institutional Review Boards and individual informed consent was waived. The study was conducted on images of 21 independent coronary segments derived from 10 patients (8 men, age 69.1 ± 9.7 years) specifically selected among those who had been exposed to both a QCA procedure due to suspected or proven coronary steno-occlusive disease, and to an IVUS examination during the same session performed to clarify ambiguous angiographic findings. Examples of ambiguous angiographic findings consist in doubts in stenosis quantification (a) due to coronary segments with diffuse atherosclerotic lesions, (b) due to the presence of a large plaque burden, or (c) when atherosclerotic lesions are located at the origin of a side branch. The 21 coronary segments (one to three per patient, length: 11.8 ± 5.0 mm) considered were obtained from seven left anterior descending arteries and three right coronary arteries.

### Quantitative Coronary Angiography

Coronary angiography was performed by a standard Judkins femoral approach. On-line QCA analysis was performed using ARTREK Quantum IC (Image Communication System, Inc., Sunnyvale, CA, USA). QCA images were recorded by using a monoplane X-ray angiogram (AXIOM-Artis, Siemens, Germany). The contrast cine-angiography (resolution: 960 × 960 pixels; frame rate: 25 frames/s) was acquired in multiple angles and saved in DICOM for off-line analyses. The angle associated with an optimal separation of the coronary region of interest from surrounding vessels was chosen. The outer diameter of the contrast-filled catheter was used for calibration. The conversion from pixel size to millimeter was performed by using scaling factors obtained by DICOM metadata.

### Active Connection Matrix Systems (ACMs): Procedures to Identify ACMs Suitable for Reprocessing QCA Images

ACMs are a collection of unsupervised artificial adaptive systems designed and developed by Semeion, Research Centre of Sciences of Communication (Rome, Italy) (11–13) [Patents: (14–17)]. The mathematical background of ACMs has been previously described (18).

**Figure 1:**
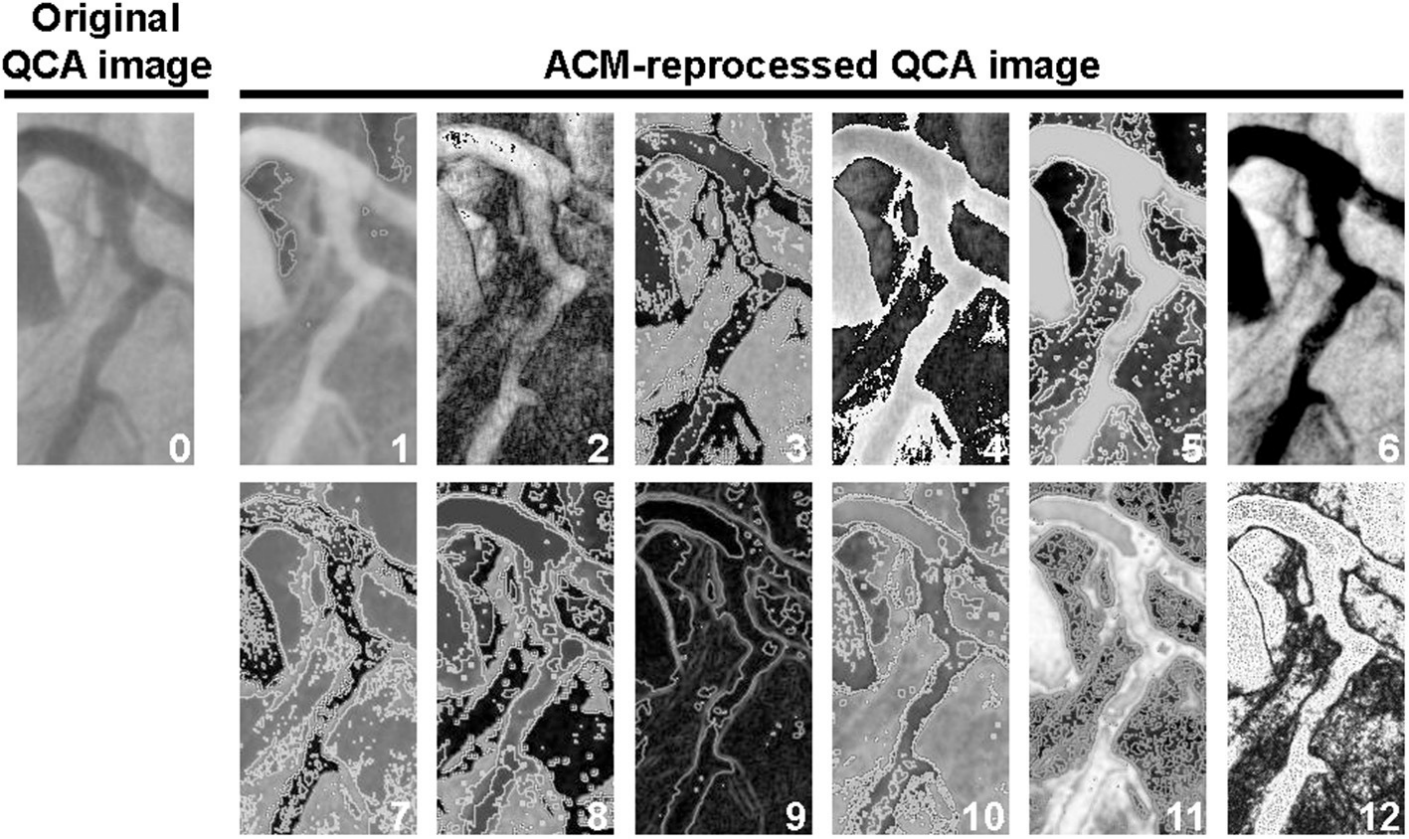
Example of one original image (image “0”) and 12 ACM-reprocessed QCA images survived to the automatic screening procedure described in the Supplementary Material. For the identification of the elective ACM/CRITERIA combination, the images survived to the automatic screening procedure were then exposed to a final visual selection. In this particular example, image “5” was considered to be the best one, because it was the only one in which, in addition to the lumen, a potential and unambiguous silhouette of the arterial wall thickness could also be recognized. Indeed, in images 1, 2, 4, 6, 9, and 12, the arterial wall thickness has not been grasped at all; images 3, 7, 8, 10, and 11 were, instead, characterized by an excessive noise.

Each ACM may reprocess an image according to different “CRITERIA” (learning laws, weights functions, pixels functions, classes, actors, dynamics, and post-processing types). Moreover, each reprocessing procedure may run with a different number of computational iterative cycles, thus generating a huge number of reprocessed images that may contain, or not, information unnoticeable in the original image. Using all possible combinations of ACMs and CRITERIA (each one run with 1, 3, 5, 10, 20, 50, 100, 150, 250, and 300 computational iterative cycles, arbitrarily set up), each image is reprocessed in ≈21.000 different ways (12 examples are shown in Figure 1). The time required to reprocess a single QCA image with one of the selected combinations of ACM and CRITERIA is in the order of 2 s.

The *a priori* identification of the ACMs/CRITERIA combinations actually able to produce additional features on QCA images is not possible. Therefore, in the initial phase of the project, we implemented a semi-automatic selection procedure to identify *a posteriori* the ACMs/CRITERIA combinations able to produce images containing anatomical structures unnoticeable in the original image. The selection procedure was run on all the 21 images of coronary segments considered. This yielded 441,000 ACM-reprocessed QCA images (21,000 × 21) for the *a posteriori* selection. Subsequently, to identify the ACMs/CRITERIA combinations able to generate reprocessed images containing new information, a high-throughput quantitative texture analysis, based on a seven-step sequential selection procedure, was developed in-house by using MATLAB R2014b (MathWorks, Inc., Natick, MA, USA).

Supplementary Figure 1 summarizes the entire procedure. A detailed technical description of the procedure to identify the ACMs/CRITERIA combinations is available on request. Details on measurements performed on both original QCA images and ACM-reprocessed QCA images are provided in Appendix 1 (Supplementary Material), also containing Supplementary Figure 2, which shows how measurements have been used to rebuild lumen and arterial wall silhouettes derived from both original and ACM-reprocessed QCA images. At the end of the semi-automatic selection procedure, one “elective ACMs/CRITERIA combination” and two “second-choice ACMs/CRITERIA combinations” were identified. The whole procedure takes about 10 min. The elective ACMs/CRITERIA combination was able to grasp potentially significant new arterial wall features in 19 out of the 21 (90.5%) coronary segments considered. In the remaining two arterial segments, where the elective ACMs/CRITERIA combination failed, new arterial features were grasped by the two second-choice ACMs/CRITERIA combinations. Being able to grasp new morphological information on the edges of the arterial silhouette, these three ACM combinations were considered suitable for a proof-of-concept study, and used to assess whether the new morphological features retrieved were artifacts or real arterial morphological structures. To this aim, a qualitative and quantitative validation of arterial features grasped by ACMs on QCA images was performed by a head-to-head comparison with IVUS used as gold standard.

The elective and the two second-choice ACMs as well as the high-throughput screening procedure are the object of a specific patent (19).

### Coronary Intravascular Ultrasound

Detailed technical descriptions of IVUS procedures and measurements of lumen diameter and wall thicknesses are provided in Appendix 2 (Supplementary Material), and illustrated in Supplementary Figures 3–7. A total of 1,364 IVUS images were measured and used for geometry comparison. The number of IVUS frames measured per patient ranged from 41 to 276.

### Qualitative and Quantitative Validation of Expected (Lumen) and New (Vessel Walls) Morphological Structures

The qualitative validation of the anatomical structures identified in ACM-reprocessed QCA images was performed by visually comparing the longitudinal silhouettes of lumen and/or arterial wall grasped by this image modality with those detected on the original QCA images and/or IVUS images (Supplementary Figures 8–18).

**Figure 2:**
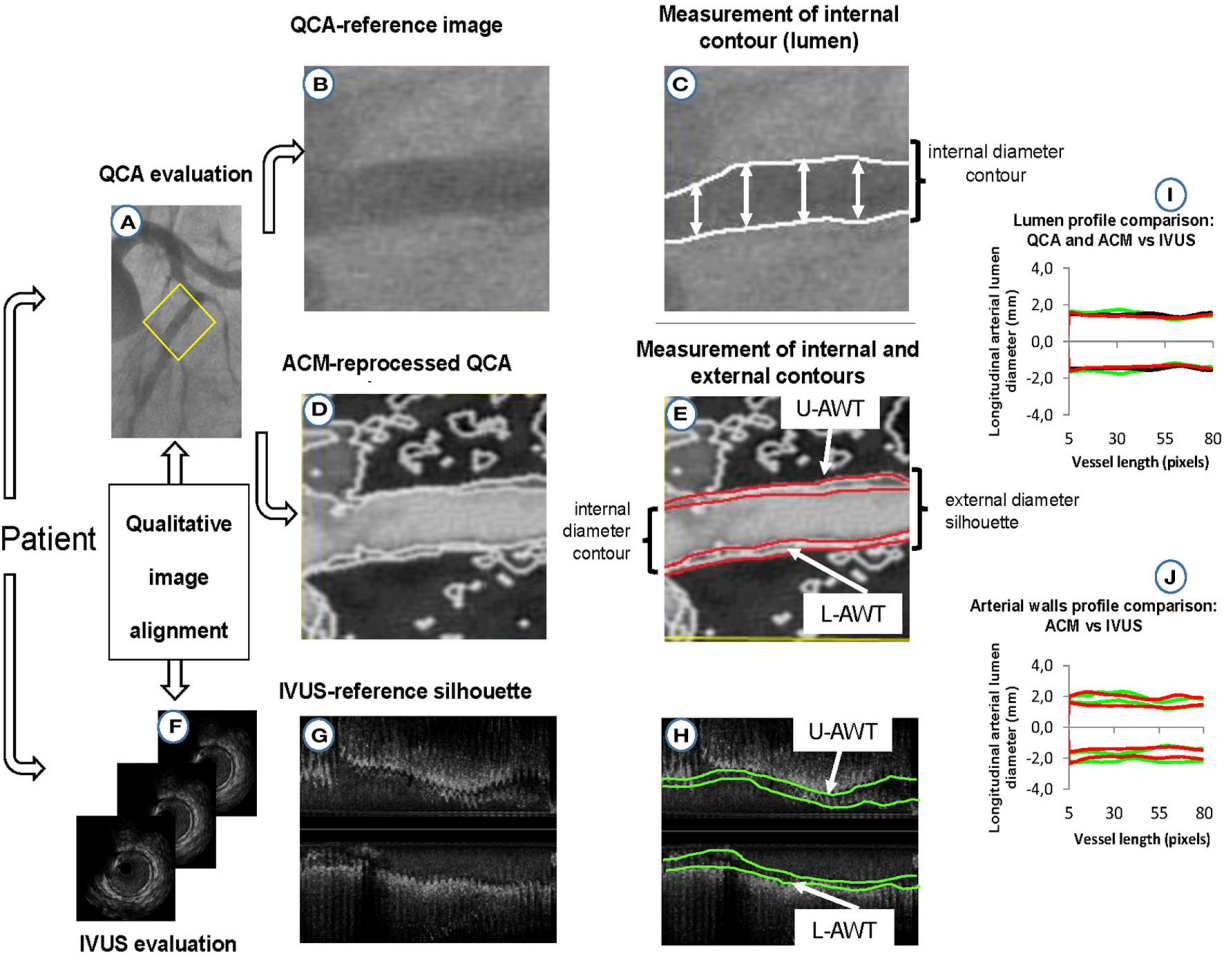
Protocol for validation of lumen and arterial wall silhouettes derived from ACM-reprocessed QCA images. **(A)** Original QCA image; **(B)** crop of the original QCA image; **(C)** lumen-diameter measured on the crop of the original QCA image; **(D)** ACM-reprocessed QCA image; **(E)** lumen (inner red lines) and adventitial (outer red lines) longitudinal contours of the artery reprocessed by ACMs. The distances between red lines in the upper and lower part of the image are supposed to be the upper (U-AWT) and lower (L-AWT) arterial wall thicknesses; **(F)** IVUS images (gold standard); **(G)** IVUS measurement converted in bi-dimensional longitudinal lumen and walls silhouettes; **(H)** lumen (inner green lines) and adventitial (outer green lines) contours of IVUS longitudinal silhouettes. The distances between the green lines in the upper and lower part of the image are the upper (U-AWT) and lower (L-AWT) arterial wall thicknesses; **(I)** qualitative concordance between lumen silhouettes derived from original QCA (black silhouettes), ACM-reprocessed QCA images (red silhouettes), and IVUS images (green silhouettes); **(J)** qualitative concordance between the arterial wall thicknesses derived from ACM-reprocessed QCA images (red silhouettes) and IVUS images (green silhouettes).

For quantitative validation, the longitudinal silhouettes of the coronary lumen and arterial walls (whenever visible) obtained with the three image modalities (original QCA images, ACM-reprocessed QCA images, and IVUS images) were fractionated in portions of 1-mm length. The minimal luminal diameter (Min-LD), the maximal luminal diameter (Max-LD), and the maximal stenosis, calculated by considering the total segment length, were also determined in each of the 21 coronary segments considered. The percent stenosis was calculated as (1 – Min-LD/Max-LD) × 100. Figure 2 summarizes the validation protocol. Max-LD, used as reference in the formula for stenosis assessment, was calculated as the mean of two diameters (measured 5 mm proximally and distally from the plaque that produces the Min-LD) to correct for vessel tapering.

### Statistical Analysis

Between-methods agreements and biases were evaluated by using Bland–Altman analysis (20). The accuracies of QCA-vs.-IVUS and ACM-vs.-IVUS were compared in absolute terms (i.e., regardless of whether they underestimate or overestimate the IVUS value) by considering the absolute value of the Bland–Altman biases. To this aim, in case of discordance in the biases direction (i.e., one bias positive and one negative), the signs of the differences vs. IVUS of the variable associated to the negative bias have been changed. The agreement of QCA and ACM-reprocessed QCA vs. IVUS was also assessed by computing absolute differences. In order to take into account the within-subject and within-coronary-segment correlation of the multiple measurements, biases and absolute differences were analyzed by Generalized Estimating Equations (GEEs). Due to non-normal distribution, results for absolute differences were confirmed by Wilcoxon signed-rank test. Significance was set at *p* < 0.05.

## Results

### Qualitative Between-Methods Agreement in Coronary Lumen and Arterial Wall Silhouettes

The qualitative concordance of the shape of lumen and arterial wall silhouettes derived by ACM-reprocessed QCA images and IVUS images of all the 21 segments considered are shown in Supplementary Figures 8–18. Each figure shows (1) the original QCA image (Panel A), (2) the same QCA image reprocessed with ACMs, where it is possible to appreciate the new features supposed to be the arterial walls (Panel B), and (3) the same image reporting also the IVUS-derived arterial wall silhouettes (green silhouettes of Panel C). All figures show that, from a qualitative point of view, the new features grasped by ACMs mirror well the arterial wall silhouettes derived by IVUS.

The descriptive measures of the 21 coronary segments obtained from “original QCA images,” “ACM-reprocessed QCA images,” and “longitudinal silhouette of IVUS images” are shown in Table 1.

**Table 1:** Measurements on the 21 coronary segments used to assess the between-methods agreement.

	**Measurements on**		
	**Original QCA images**	**ACM-reprocessed QCA images**	**Longitudinal silhouette of IVUS images**
Mean lumen diameter, mm	3.60 ± 0.76	2.76 ± 0.74	3.01 ± 0.63
Minimal luminal diameter, mm	2.71 ± 0.48	1.67 ± 0.54	2.12 ± 0.43
Percent stenosis, %^*^	14.6 ± 10.2	23.7 ± 18.9	18.2 ± 14.7
Mean U-AWT, mm	Not applicable	0.72 ± 0.12	0.68 ± 0.24
Mean L-AWT, mm	Not applicable	0.69 ± 0.14	0.58 ± 0.12
Maximal U-AWT, mm	Not applicable	1.04 ± 0.23	1.05 ± 0.38
Maximal L-AWT, mm	Not applicable	1.07 ± 0.22	0.88 ± 0.22

*Continuous data are mean ± SD*.

* *IVUS percent stenoses are calculated on longitudinal view. U-AWT, upper arterial walls thickness; L-AWT, lower arterial walls thickness*.

### Quantitative Between-Methods Agreement in the Percent of Coronary Stenosis

**Figure 3:**
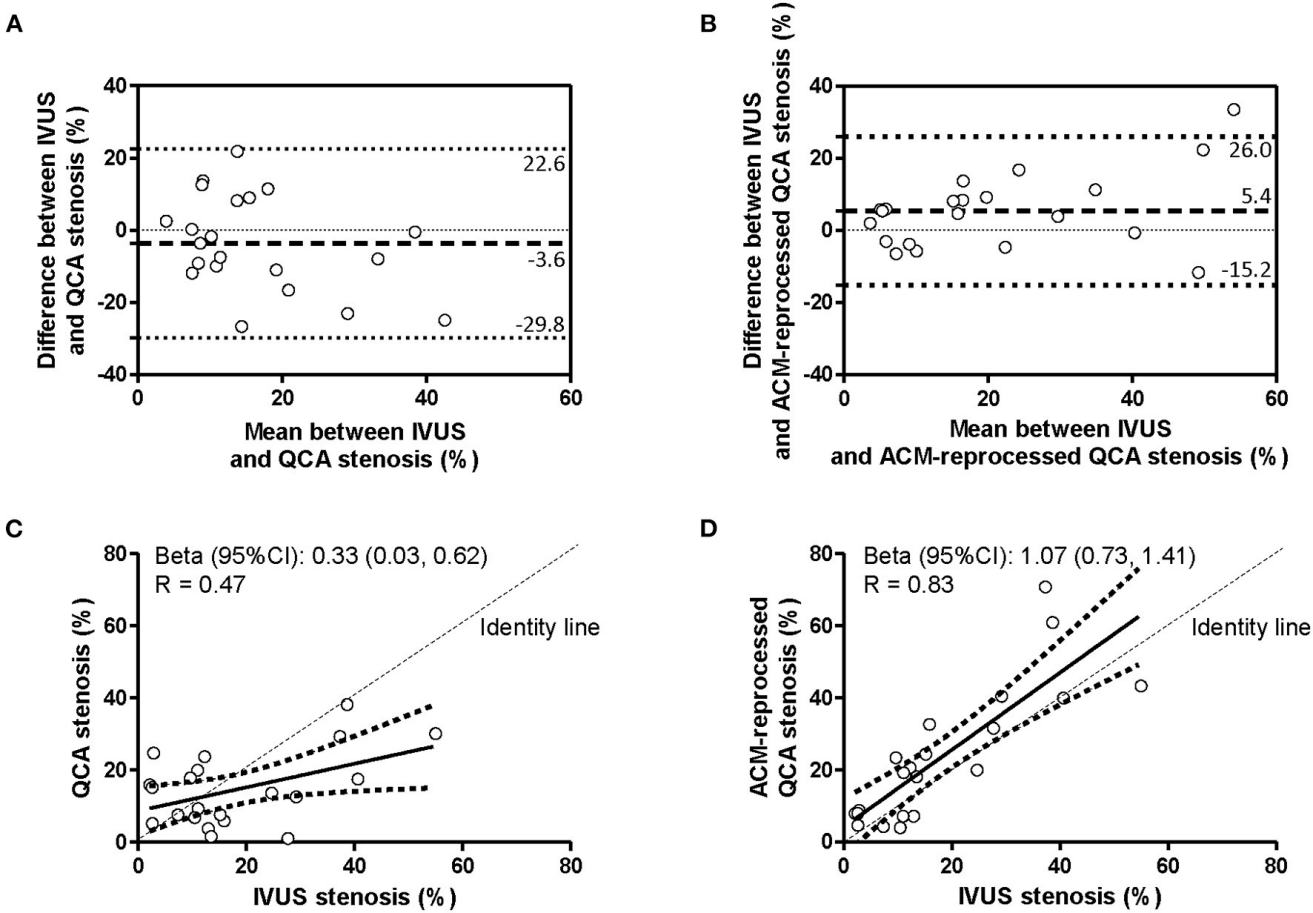
Agreement in measurement of percent of stenosis. Bland–Altman plots **(A,B)** and linear regression analyses **(C,D)** assessing the agreement between the percent of stenosis derived by IVUS and that derived by original QCA images **(A,C)** or by ACM-reprocessed QCA images **(B,D)**. Continuous lines in the scatter plots indicate the regression line and 95% CI.

The agreement in the percent coronary stenosis was assessed by considering each coronary segment for the whole length. Bland–Altman plots (Figures 3A,B and Table 2) revealed no significant bias differences vs. IVUS measurements when “original” or “ACM-reprocessed QCA images” were considered [mean difference between absolute biases (95% CI) = −1.84 (−9.44, 5.76) mm, *p* = 0.63 by GEE]. Accordingly, also the delta absolute differences were not significantly different. By contrast, the correlation coefficient (Figures 3C,D) improved from 0.47 to 0.83 when “original QCA images” or “ACM-reprocessed QCA images” were, respectively, plotted against IVUS.

**Table 2:** Morphologic measurements of the pooled vessel lumen stenosis, lumen diameter, and vessel wall thickness among measurements at original QCA, ACM-reprocessed QCA, and IVUS.

	**QCA vs. IVUS**	**ACM vs. IVUS**
**Lumen stenosis (%;** ***n*** **=** **21)**		
Bias between methods	−3.60 (−9.17, 1.98)	5.44 (1.05, 9.83)^*^
Delta absolute biases (ACM – QCA)^†^	−1.84 (−9.44, 5.76)	
Absolute differences between methods	10.00 (7.50, 13.72)^§^	5.92 (4.61, 11.24)^§^
Delta absolute differences (ACM – QCA)	−2.26 (−7.28, 2.76)	
**Lumen diameter (mm;** ***n*** **=** **263)**		
Bias between methods	0.58 (0.45, 0.72)^#^	−0.25 (−0.37, −0.14)^#^
Delta absolute biases (ACM – QCA)^†^	−0.33 (−0.54, −0.13)^**^	
Absolute differences between methods	0.49 (0.45, 0.55)^§^	0.28 (0.23, 0.34)^§^
Delta absolute differences (ACM – QCA)	−0.23 (−0.40, −0.07)^**^	
**Wall thickness (mm;** ***n*** **=** **526)**		
Bias between methods	Not applicable	0.09 (0.03, 0.14)^**^
Absolute differences between methods	Not applicable	0.16 (0.14, 0.18)^§^

*Biases are calculated as recommended by Bland–Altman (mean of between readings relative differences)*.

† *As the signs of the two biases were discordant, delta absolute biases were calculated by changing the signs of the differences between ACM and IVUS readings (see Materials and Methods). Biases, delta biases, and delta absolute differences are reported as mean (95% CI of the mean); absolute differences are reported as median (95% CI of the median). p-values of paired comparisons were computed by Generalized Estimating Equations (GEE), accounting for within-patient and within-segment measures correlations*.

* * <0.05*;

** * <0.01*;

# * <0.001*;

§ *significance not reported (as, by definition, absolute differences are always >0)*.

### Quantitative Between-Methods Agreement in Lumen Diameter Assessment

For the quantitative between-methods agreement in lumen diameter assessment, the longitudinal silhouettes of the coronary segments, obtained with the three imaging modalities (Supplementary Figure 7) and fractionated in portions of 1-mm length, generated 263 values for lumen comparison.

**Figure 4:**
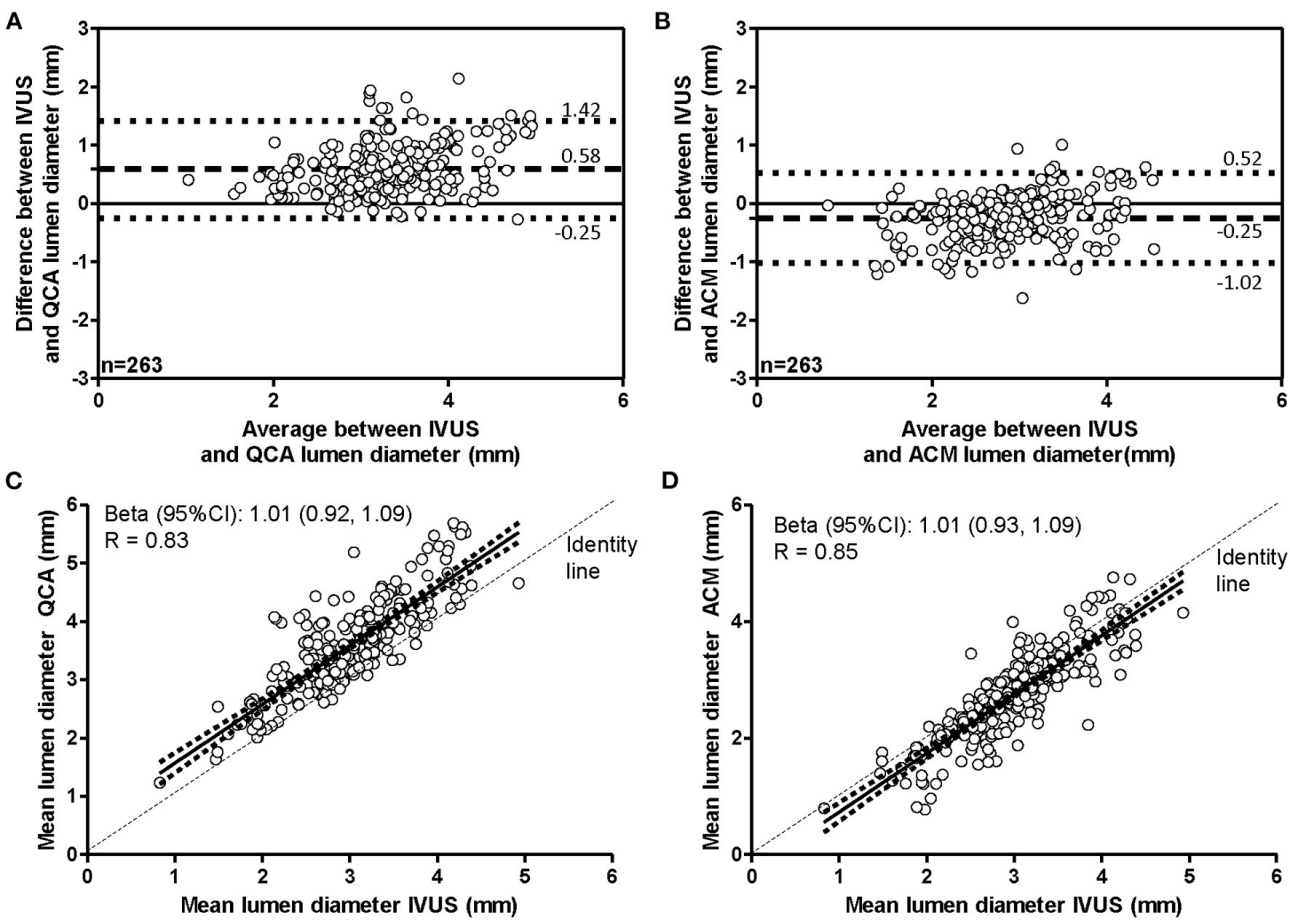
Agreement in measurement of lumen diameters. Bland–Altman plots **(A,B)** and linear regression analyses **(C,D)** assessing the agreement between the lumen diameters derived by IVUS and that derived by original QCA images **(A,C)** or by ACM-reprocessed QCA images **(B,D)**. Continuous lines in the scatter plots indicate the regression line and 95% CI.

Bland–Altman plots (Figure 4A) revealed a bias of 0.58 mm when lumen diameter measured on original QCA images was compared with that measured on IVUS images (gold standard). According to GEE analysis, such bias was statistically significant (*p* < 0.001; Table 2). The correlation coefficient was 0.83, and the slope very close to 1 (Figure 4C).

When IVUS-derived lumen diameters were compared with those obtained on ACM-reprocessed QCA images (Figure 4B), the bias was still significant (*p* < 0.001 by GEE); however, both the bias and the absolute differences between methods were significantly smaller (−0.25 and 0.28 mm, respectively) than that observed between QCA images and IVUS images (0.58 and 0.49 mm, respectively; Table 2). Both the mean difference between absolute biases (−0.33 mm) and the delta absolute difference (−0.23 mm) were also significant (*p* = 0.0014 and *p* = 0.0063, respectively). The correlation coefficient (0.85) and the slope (1.01) remained almost the same (Figure 4D).

### Quantitative Between-Methods Agreement in Arterial Wall Thickness

**Figure 5:**
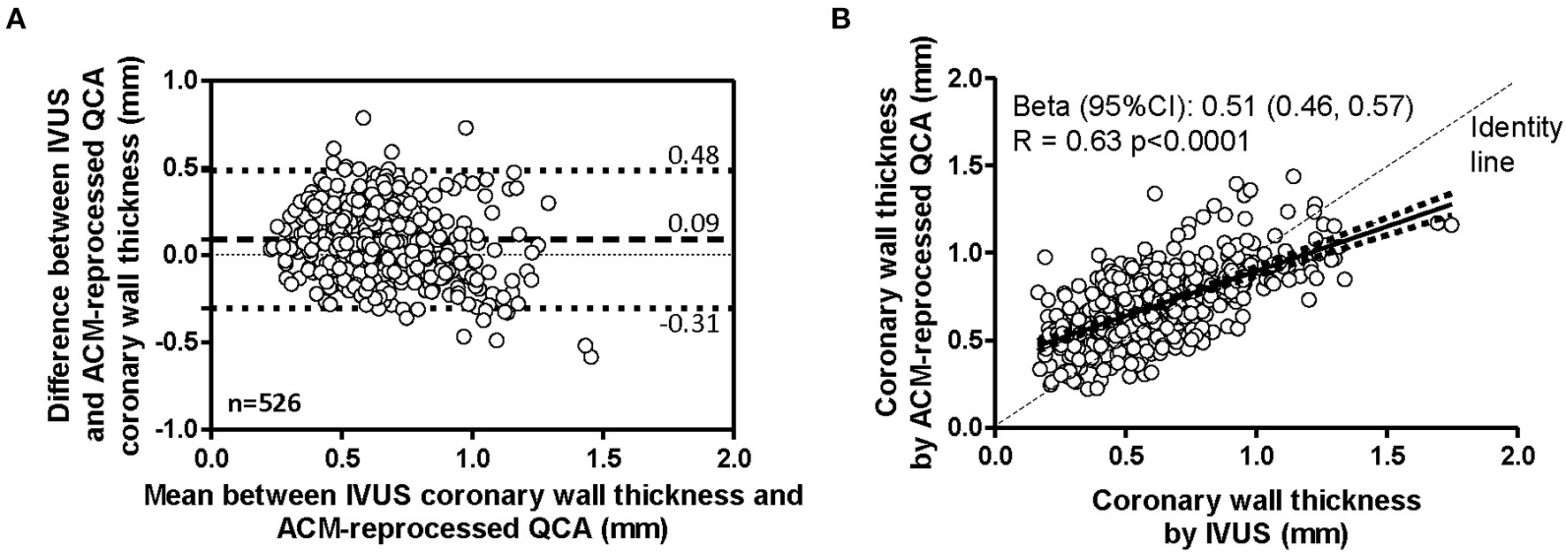
Agreement in measurement of coronary wall thicknesses. Bland–Altman plot **(A)** and linear regression analysis **(B)** assessing the agreement between the arterial wall thicknesses derived by IVUS and by ACM-reprocessed QCA images. In the scatter plot, continuous lines indicate the regression line and 95% CI.

Considering the 21 coronary segments in their entire length, the average arterial wall thickness was 0.70 ± 0.21 mm (range 0.22, 1.44 mm) when measured on ACM-reprocessed QCA images and 0.61 ± 0.25 mm (range 0.16, 1.75 mm) when measured on longitudinal reconstructed IVUS arterial wall silhouettes. Supplementary Figures 8–18 show the concordance of the shape of arterial wall silhouettes from a qualitative point of view. As done for the lumen, also for the quantitative between-methods agreement in the assessment of arterial walls thickness, the longitudinal silhouettes of coronary segments (Figure 5) were fractionated in portions of 1-mm length, generating 263 values for the upper (U-AWT) and 263 values for the lower (L-AWT) arterial wall thicknesses. Both the Bland–Altman plots (Figure 5A) and the regression analysis (Figure 5B) revealed that the arterial wall thickness grasped by ACMs on QCA images mirrors well the measures derived by IVUS images (gold standard). Indeed, the bias between the measures obtained with IVUS and ACMs, although significant in the GEE analysis (*p* < 0.01), was very small [0.09 mm, 95% CI (0.03, 0.14); Table 2] and the correlation between the two imaging modalities was fairly good (*r* = 0.63; *p* < 0.0001; Figure 5B).

## Discussion

Correlation between standard QCA and IVUS measurements of the lumen has been shown to be excellent just in normal coronary arteries, but only moderate when diseased arteries were considered (21). Beyond corroborating such moderate correlation, in this proof-of-concept study we have shown that, when QCA images are reprocessed with ACMs, measurements of lumen diameter improve and, compared with IVUS data, are better than those measured on standard QCA images. Furthermore, this is the first study to show that ACMs allow grasping additional arterial features that mirror well the arterial wall profile derived by IVUS. This is a novel finding as, so far, arterial wall information could be accurately detected only by IVUS (7, 22–24).

The accurate assessment of lesion severity is crucial for decision-making in the cardiac catheterization laboratory. Indeed, regardless of whether the lesion protrudes into the lumen or not, the underestimation of the disease severity would leave critical lesions untreated, while an overestimation would lead to unnecessary revascularization procedures that could cause coronary restenosis for catheter-induced injury (25, 26). Yet, QCA assessment is still visual and highly observer-dependent, and it is known that the visual interpretation of standard coronary arteriograms cannot visualize eccentric plaques (27) and is a poor predictor of the physiological importance of coronary stenosis (28). Moreover, although pivotal to evaluate plaques protruding into the lumen, standard QCA is unsuitable for plaques developed extensively into the arterial wall but negligibly protruding into the lumen, due to the phenomena of outward arterial remodeling (29, 30). As a consequence, such “invisible-to-QCA” lesions are neglected in clinical practice, despite evidences of an association between non-protruding plaques and risk of events (31). Plausibly, a timely and accurate identification/quantification of these non-flow-limiting lesions through ACM-reprocessed QCA might help to improve personalization of preventive interventions.

The ACM approach has several advantages even when compared to IVUS. First, it evades the potential risks of minor (e.g., endothelial damage) or major (e.g., arterial dissection) complications of the intra-coronary passage of a transducer. Second, though not assessed in this study, it may provide information about coronary segments not reached by the IVUS transducer. Accordingly, a reprocessing tool for the assessment of regional coronary arterial geometry that (1) does not require additional diagnostic procedures, (2) is able to visualize not protruding lesions, and (3) is more objective, consistent, and widely available not only to experts but also to less trained personnel has a high potential to increase the prognostic performance of QCA and would be of great interest in both clinical and research setting.

The results here presented have to be interpreted with caution. Indeed, the study being just a “proof of concept,” we do not want to claim that the ACM-reprocessed QCA images can be considered as a replacement of IVUS, but rather that, adopting these systems, there is today the concrete possibility to improve the measurement accuracy of arterial conventional features from QCA images and, overall, to grasp arterial wall information that, till now, were completely invisible in the original images.

The study has some limitations. First, it does not include a comparison with a standard of reference like pathology; however, the IVUS (our gold standard) was previously validated against histology (32, 33) and has often been used as the reference standard of coronary vessel wall evaluation. Second, our results refer to coronary segments free of lumen-protruding plaques, and further research, in coronary segments with such characteristics, is warranted. Third, the sample size is small. Regardless that, with proof-of-concept studies, a small sample is almost the norm (34, 35), it has to be underlined that we have analyzed multiple segments per subject (*n* = 21 segments for 10 patients) and multiple (1 mm by 1 mm) sub-segments per segment (for a total of 263 sub-segments analyzed). With such a number of comparisons, we believe that our results give a good impression about possibilities and limitations of ACM-reprocessing of QCA compared with IVUS. Finally, it has to be underlined that this is just an initial step toward the assessment of coronary walls from QCA images. Further studies with larger sample sizes and training-and-testing procedures have to be performed for a more comprehensive assessment of the potentialities of this novel vessel wall reprocessing approach.

In conclusion, this proof-of-concept study shows that the use of ACMs to reprocess conventional QCA images markedly improves the accuracy in lumen diameter and allows grasping additional features, invisible on the original images, which mirror well the arterial wall profile derived by IVUS. If validated in larger samples, the approach here proposed might have a great interest and transferability into the clinical practice and suggests a potential role of this test as a first-line diagnostic modality, i.e., prior to coronary IVUS, for excluding the presence of hidden plaques in non-obstructive coronary disease.

## Data Availability Statement

The raw data supporting the conclusions of this article will be made available by the authors, without undue reservation.

## Ethics Statement

The studies involving human participants were reviewed and approved by Comitato Etico IRCCS—Istituto Europeo di Oncologia e Centro Cardiologico Monzino—Via Ripamonti 435 20141 Milano MI. Written informed consent for participation was not required for this study in accordance with the national legislation and the institutional requirements. The ANN-IDA is a proof-of-concept retrospective study. In agreement with internal procedures in force at the Centro Cardiologico Monzino, also approved by the local Institutional Review Boards, individual informed consent was waived. The study has been performed by using archive images collected between 2012 and 2013 and no risk for the patients' privacy has been identified. For this reason, patient consent was waived.

## Author Contributions

MA, MB, ET, and DB: conceptualization. MA, MB, GMas, GMau, EG, BF, AR, DS, and DC: methodology. MA, MB, GMas, and DB: validation. MA, FV, and DB: formal analysis. MA, MB, GMas, GMau, BF, AR, DS, DC, CF, and AB: investigation. MA, FV, ET, and DB: data curation. MA, MB, and DB: writing—original draft preparation and project administration. MA, MB, GMas, GMau, EG, BF, AR, DS, DC, CF, AB, FV, ET, and DB: writing—review and editing. MB, ET, and DB: supervision. ET: funding acquisition. All authors have read and agreed to the published version of the manuscript.

## Funding

This research was funded by the Italian Ministry of Health, Rome, Italy (RC2011 ID-2102343, RC2012 ID-2351878, RC 2013 ID-2600725, RC 2014 ID-2607406, RC2015 ID-2613071, RC2016 ID-2622813, RC2017 ID-2631162, RC2018 ID-2634534, and RC2019 ID-2755481).

## Conflict of Interest

Centro Cardiologico Monzino and Semeion have a research patent-licensing arrangement with Semeion Center concerning the use of ACM systems. DB, MA, ET, and MB are co-inventors of a patent application (Italian patent Application Number IT201800001656A, published on July 23, 2019 and retrievable at https://www.uibm.gov.it/bancadati/Number_search/type_url?type=wpn with the code: 102018000001656; International patent Application Number IB2019050520W, published on August 1, 2019 and retrievable at https://patents.google.com/patent/WO2019145849A1/en and at https://worldwide.espacenet.com/patent/search/family/062089850/publication/WO2019145849A1?q=102018000001656). The remaining authors declare that the research was conducted in the absence of any commercial or financial relationships that could be construed as a potential conflict of interest.

## Publisher's Note

All claims expressed in this article are solely those of the authors and do not necessarily represent those of their affiliated organizations, or those of the publisher, the editors and the reviewers. Any product that may be evaluated in this article, or claim that may be made by its manufacturer, is not guaranteed or endorsed by the publisher.

## References

[1] Nishioka T Amanullah AM Luo H Berglund H Kim CJ Nagai T. Clinical validation of intravascular ultrasound imaging for assessment of coronary stenosis severity: comparison with stress myocardial perfusion imaging. J Am Coll Cardiol. (1999) 33:1870–8. 10362187 10.1016/s0735-1097(99)00100-x

[2] Jensen LO Thayssen P Mintz GS Egede R Maeng M Junker A. Comparison of intravascular ultrasound and angiographic assessment of coronary reference segment size in patients with type 2 diabetes mellitus. Am J Cardiol. (2008) 101:590–5. 10.1016/j.amjcard.2007.10.020 18308004

[3] Bourantas CV Tweddel AC Papafaklis MI Karvelis PS Fotiadis DI Katsouras CS. Comparison of quantitative coronary angiography with intracoronary ultrasound. Can quantitative coronary angiography accurately estimate the severity of a luminal stenosis? Angiology. (2009) 60:169–79. 10.1177/0003319708317338 18508852

[4] Poyet R Cuisset T Bali L Quilici J Lambert M Bonnet JL. Coronary wall characteristics after myocardial infarction without significant coronary angiographic lesion: an intravascular ultrasound study. Acta Cardiol. (2010) 65:627–30. 10.2143/AC.65.6.2059858 21302667

[5] Hermiller JB Tenaglia AN Kisslo KB Phillips HR Bashore TM Stack RS. *In vivo* validation of compensatory enlargement of atherosclerotic coronary arteries. Am J Cardiol. (1993) 71:665–8. 8447262 10.1016/0002-9149(93)91007-5

[6] American College of Cardiology Clinical Expert Consensus Document on Standards for Acquisition Measurement and Reporting of Intravascular Ultrasound Studies (IVUS). A report of the American College of Cardiology Task Force on Clinical Expert Consensus Documents developed in collaboration with the European Society of Cardiology endorsed by the Society of Cardiac Angiography and Interventions. Eur J Echocardiogr. (2001) 2:299–313. 10.1053/euje.2001.0133 11908481

[7] Nicholls SJ Tuzcu EM Sipahi I Schoenhagen P Nissen SE. Intravascular ultrasound in cardiovascular medicine. Circulation. (2006) 114:e55–9. 10.1161/CIRCULATIONAHA.106.637942 16864731

[8] Castellano G Bonilha L Li LM Cendes F. Texture analysis of medical images. Clin Radiol. (2004) 59:1061–9. 10.1016/j.crad.2004.07.008 15556588

[9] Dennie C Thornhill R Sethi-Virmani V Souza CA Bayanati H Gupta A. Role of quantitative computed tomography texture analysis in the differentiation of primary lung cancer and granulomatous nodules. Quant Imaging Med Surg. (2016) 6:6–15. 10.3978/j.issn.2223-4292.2016.02.01 26981450 PMC4775240

[10] Gastounioti A Conant EF Kontos D. Beyond breast density: a review on the advancing role of parenchymal texture analysis in breast cancer risk assessment. Breast Cancer Res. (2016) 18:91. 10.1186/s13058-016-0755-8 27645219 PMC5029019

[11] Buscema M Catzola L Grossi E. Images as active connection matrixes: the J-Net system. Int J Intell Comput Med Sci Image Proc. (2007) 2:27–53. 10.1080/1931308X.2008.10644150

[12] Buscema P. Sistemi ACM e Imaging Diagnostico. Le immagini mediche come matrici attive di connessioni [ACM Systems and Diagnostic Imaging. Medical Images as Active Connections Matrices, in Italian]. Milan: Springer Verlag (2006).

[13] Buscema M Passariello R Grossi E Massini G Fraioli F Serra G. J-Net: an adaptive system for computer-aided diagnosis in lung nodule characterization. In: Tastle WJ, editor. Data Mining Applications Using Artificial Adaptive Systems. New York, NY: Springer Science + Business Media (2013). p. 25–61.

[14] Buscema PM. European Patent “An Algorithm for Recognizing Relationships Between Data of a Database and a Method for Image Pattern Recognition Based on the Said Algorithm” (Active Connection Matrix -ACM). Owner: Semeion. EPO no. 1508872. Application No. 03425559.6 (2003).

[15] Buscema PM. Neural Network for Processing Arrays of Data With Existent Topology, Such as Images and Application of the Network. Owner: Semeion US Patent 20070233624A1 (2007).

[16] Buscema PM. Neural Network for Processing Arrays of Data With Existent Topology, Such as Images and Application of the Network. Owner: Semeion US Patent US7877342B2 (2011).

[17] Buscema M. ACM Active Connection Matrix, SEMEION Software n. 30, ver. 12.5. Rome (2003–2009).

[18] Buscema M. Four new adaptive systems for four medical applications; part 1. In: NAFIPS 2008 – 2008 Annual Meeting of the North American Fuzzy Information Processing Society. New York, NY (2008). p. 1–6.

[19] Baldassarre D Amato M Tremoli E Buscema M. Method for Processing Images and Method for Determining Quantitative and/or Qualitative Characteristics of Objects Reproduced in an Image, System for Processing Images and System for Determining Quantitative and/or Qualitative Characteristics of the Objects Reproduced in an Image Owner: Centro Cardiologico Monzino and Semeion. Italian patent Application number IT201800001656A (2018). Retrievable at: https://www.uibm.gov.it/bancadati/Number_search/type_url?type=wpn with the code: 102018000001656 (accessed July 23, 2019); International patent Application number IB2019050520W. Retrievable at: https://patents.google.com/patent/WO2019145849A1/en; https://worldwide.espacenet.com/patent/search/family/062089850/ publication/WO2019145849A1?q=102018000001656 (accessed August 1, 2019).

[20] Bland JM Altman DG. Measuring agreement in method comparison studies. Statist Methods Med Res. (1999) 8:135–60. 10.1177/096228029900800204 10501650

[21] De Scheerder I De Man F Herregods MC Wilczek K Barrios L Raymenants E. Intravascular ultrasound versus angiography for measurement of luminal diameters in normal and diseased coronary arteries. Am Heart J. (1994) 127:243–51. 8296690 10.1016/0002-8703(94)90110-4

[22] Swallow RA Court IA Calver AL Curzen NP. The limitations of coronary angiography: identification of a critical coronary stenosis using intravascular ultrasound. Int J Cardiol. (2006) 106:123–5. 10.1016/j.ijcard.2004.11.032 16321677

[23] Yamagishi M Hosokawa H Saito S Kanemitsu S Chino M Koyanagi S. Coronary disease morphology and distribution determined by quantitative angiography and intravascular ultrasound–re-evaluation in a cooperative multicenter intravascular ultrasound study (COMIUS). Circ J. (2002) 66:735–40. 10.1253/circj.66.735 12197597

[24] Mintz GS Painter JA Pichard AD Kent KM Satler LF Popma JJ. Atherosclerosis in angiographically “normal” coronary artery reference segments: an intravascular ultrasound study with clinical correlations. J Am Coll Cardiol. (1995) 25:1479–85. 7759694 10.1016/0735-1097(95)00088-l

[25] Luo H Nishioka T Eigler NL Forrester JS Fishbein MC Berglund H. Coronary artery restenosis after balloon angioplasty in humans is associated with circumferential coronary constriction. Arterioscler Thromb Vasc Biol. (1996) 16:1393–8. 8911279 10.1161/01.atv.16.11.1393

[26] Mintz GS Popma JJ Pichard AD Kent KM Satler LF Wong C. Arterial remodeling after coronary angioplasty: a serial intravascular ultrasound study. Circulation. (1996) 94:35–43. 8964115 10.1161/01.cir.94.1.35

[27] Rasheed Q Hodgson JM. Application of intracoronary ultrasonography in the study of coronary artery pathophysiology. J Clin Ultrasound. (1993) 21:569–78. 8227387 10.1002/jcu.1870210904

[28] White CW Wright CB Doty DB Hiratza LF Eastham CL Harrison DG. Does visual interpretation of the coronary arteriogram predict the physiologic importance of a coronary stenosis? New Engl J Med. (1984) 310:819–24. 10.1056/NEJM198403293101304 6700670

[29] Jegere S Narbute I Erglis A. Use of intravascular imaging in managing coronary artery disease. World J Cardiol. (2014) 6:393–404. 10.4330/wjc.v6.i6.393 24976911 PMC4072829

[30] Glagov S Weisenberg E Zarins CK Stankunavicius R Kolettis GJ. Compensatory enlargement of human atherosclerotic coronary arteries. New Engl J Med. (1987) 316:1371–5. 3574413 10.1056/NEJM198705283162204

[31] Min JK Dunning A Lin FY Achenbach S Al-Mallah M Budoff MJ. Age- and sex-related differences in all-cause mortality risk based on coronary computed tomography angiography findings results from the International Multicenter CONFIRM (Coronary CT Angiography Evaluation for Clinical Outcomes: an International Multicenter Registry) of 23,854 patients without known coronary artery disease. J Am Coll Cardiol. (2011) 58:849–60. 10.1016/j.jacc.2011.02.074 21835321

[32] Neville RF Hobson RW II Jamil Z Breitbart GB Anderson RJ Bartorelli AL. Intravascular ultrasonography: validation studies and preliminary intraoperative observations. J Vasc Surg. (1991) 13:274–82; discussion: 82–3. 1990168

[33] Wenguang L Gussenhoven WJ Zhong Y The SH Di Mario C Madretsma S. Validation of quantitative analysis of intravascular ultrasound images. Int J Card Imaging. (1991) 6:247–53. 1919067 10.1007/BF01797856

[34] Botnar RM Stuber M Kissinger KV Kim WY Spuentrup E Manning WJ. Noninvasive coronary vessel wall and plaque imaging with magnetic resonance imaging. Circulation. (2000) 102:2582–7. 10.1161/01.cir.102.21.2582 11085960

[35] Fayad ZA Fuster V Fallon JT Jayasundera T Worthley SG Helft G. Noninvasive *in vivo* human coronary artery lumen and wall imaging using black-blood magnetic resonance imaging. Circulation. (2000) 102:506–10. 10.1161/01.cir.102.5.506 10920061

