# Supplementary material for: Assessment of New Coronary Features on Quantitative Coronary Angiographic Images With Innovative Unsupervised Artificial Adaptive Systems: A Proof-of-Concept Study
Source: Front Cardiovasc Med. 2021 Oct 14;8:730626. doi: 10.3389/fcvm.2021.730626 (PMC8551448; doi:10.3389/fcvm.2021.730626)
Supplement: Supplementary file 1 [file Data_Sheet_1.PDF]

Supplementary Material

Supplementary Methods

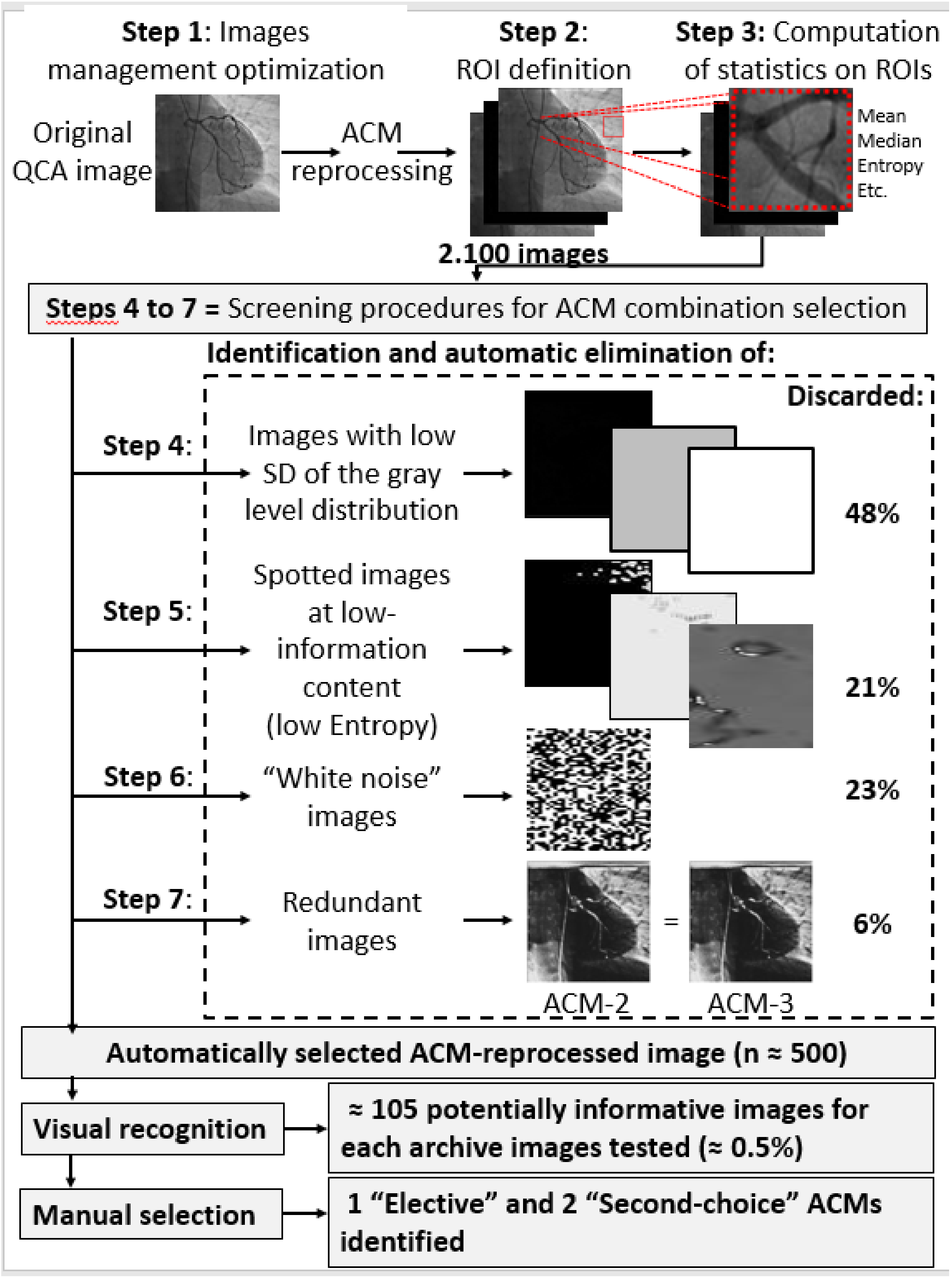

Supplementary Figure 1. Flow diagram summarizing the high-throughput screening of ACM-reprocessed images.

Appendix-1

Measurements on original QCA-images and ACM-reprocessed QCA-images: methodological details

All the images of the selected QCA cine film of each patient were used. The mean duration of the QCA cines was 2.5±0.4 seconds (range from 1.8 to 3.2 seconds). Considering the acquisition frame rate of 25 frames/sec, a total of 63±11 QCA-images per patient (25 × ≈2.5) were obtained and reprocessed with the 3 ACM set/rules identified by the ACM screening procedure. On average, the reprocessed cine film of each patient was constituted by ≈189±34 images (≈63 QCA-images × 3 ACM set/rules) for a total of 1890 images considering the 10 patients. Each one of these 1890 images were visually screened in order to select at least 10 images (one per patient) containing new morphological structures reassembling, at least potentially, the coronary arterial walls.

In each original and ACM-reprocessed QCA image, the lumen diameter of both QCA and ACM-reprocessed images was independently measured by two trained readers (A.R. with 13 years of experience and B.F. with 10 years of experience) by using a semi-automated contour edge-detection system (1). Both readers were blinded to clinical information and IVUS reports. The software automatically produces measures of the lumen diameter spaced each other by one pixel along the longitudinal axis of the artery. The same software was also used to measure, on ACM-reprocessed images, the distance between the anatomical structures supposed to be the blood-intima and media-adventitia interfaces of the upper arterial walls thickness (U-AWT) and of the lower arterial walls thickness (L-AWT).

Smoothing the QCA diameter silhouettes by a sliding windows mathematical approach

Smoothed silhouettes for arterial lumen diameters were derived from both original and ACM-reprocessed QCA measurements by using a sliding window mathematical approach.

Specifically, two “sliding windows” averaging 5 consecutive measurements of arterial lumen diameter were moved along the vessel axis by consecutive steps of one point. The first sliding window was moved from the first to the last measure of the lumen. The second was moved in the opposite direction (i.e. from the last to the first measure of the lumen). The values obtained at each step (i.e. the average of the 5-consecutive measurements) by the two sliding windows were then averaged and the average was plotted vs the length of the segment to obtain the smoothed silhouettes of the lumen (Supplementary Figure 2, left panel). Similarly, smoothed silhouettes of the anatomical structures supposed to be blood-intima and media-adventitia interfaces of the U-AWT and L-AWT from ACM-reprocessed QCA-images were also obtained (Supplementary Figure 2, right panel).

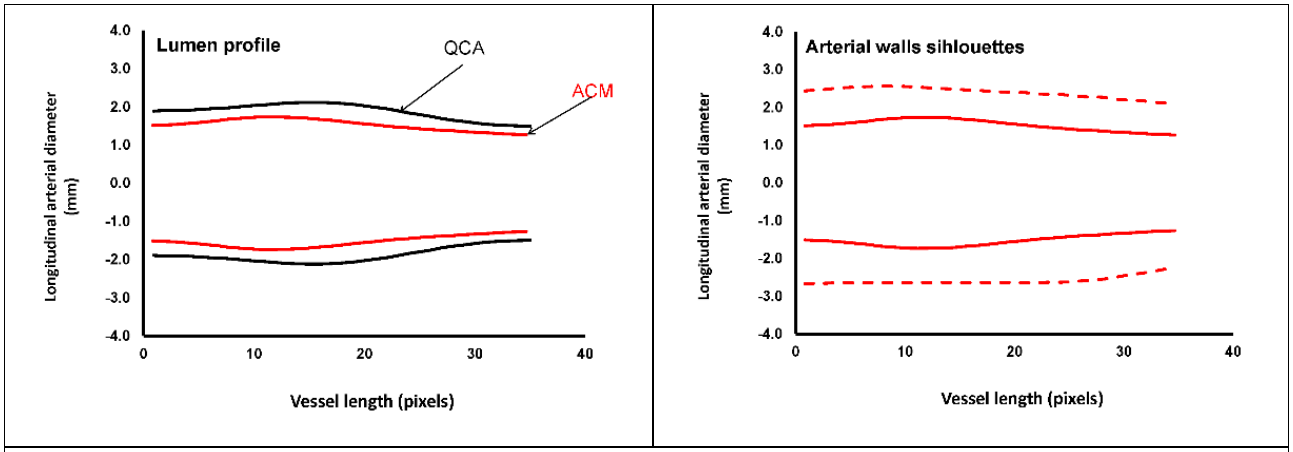

Supplementary Figure 2. Lumen and arterial wall silhouettes derived from original QCA-images and from ACM-reprocessed QCA-images. The left panel shows the lumen silhouettes obtained by original QCA-images (black line) and ACM-reprocessed QCA-images (red line). In the right panel, the distance between the solid and dotted red lines denotes the silhouettes derived from ACM-reprocessed QCA-images, which are supposed to be arterial wall thicknesses.

Appendix-2

IVUS measurements of both lumen diameter and vessel wall thickness

After intracoronary injection of nitrate (200 µg i.c.) and heparin (2.500 U i.v.), IVUS-images acquisition of the coronary region of interest (target vessel) was performed with a 30 MHz mechanical ultrasound transducer rotating at 1800 rpm. Images were acquired with a frame rate of 30 frames/sec by using a validated (2, 3) automatic pullback device withdrawal at 0.5 mm/s (axial resolution = 0.017 mm/frame) within a 3.2 F short monorail imaging catheter (CardioVascular Imaging System, Inc., Sunnyvale, CA, USA), for the entire length of the target vessel. Images of the entire pullback were saved in standard DICOM format on DVD for subsequent quantitative analysis. Also in this case, the conversion from pixel size to millimeter was performed by using scaling factors obtained by DICOM metadata.

IVUS-images were analysed off-line by a trained reader (A.R. with 15 years of experience) blinded to the QCA results, according to the standards of American College of Cardiology and European Society of Cardiology guidelines, as previously described (4). The entire set of cross-sectional IVUS-images obtained in the coronary segments investigated was visually reviewed. IVUS-images without an optimal delineation of the blood-intimal border and/or with calcifications occupying most of the vessel circumference were excluded. Among eligible images, one image every twenty was selected for measurements. In such images, the external elastic membrane cross-sectional area (EEM-CSA) and the lumen cross-sectional area (L-CSA) were traced by a semi-automatic software (5). Artefacts were corrected manually, as previously described (6, 7).

The lumen diameter, as well as the distances between the blood-intima and media-adventitia interfaces of the U-AWT and L-AWT of each cross-sectional IVUS image, were measured according to 8 angles rotated of 45° degrees around the center of luminal axis (Supplementary Figure 3).

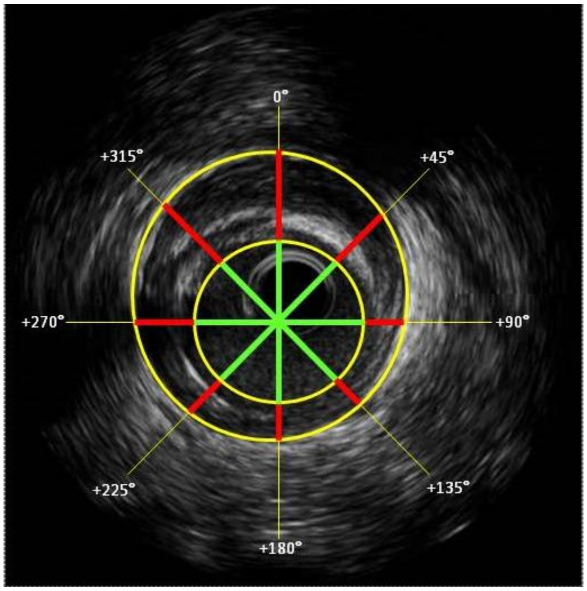

Supplementary Figure 3. Cross-sectional IVUS image showing the 8 angles where the arterial walls have been measured. The angles correspond to rotation of 45° degrees around the center of luminal axis. The top vertical point was designated arbitrarily as zero degrees.

Multiple angle measurements were done because it is impossible to know *a-priori* which IVUS slice-orientation has to be used for comparison with the lumen silhouette provided by QCA. In order to identify the most appropriate IVUS slice-orientation for such comparison, the IVUS measurements obtained in the 8 angles were converted in 8 bi-dimensional longitudinal lumen and walls silhouettes (Supplementary Figure 4).

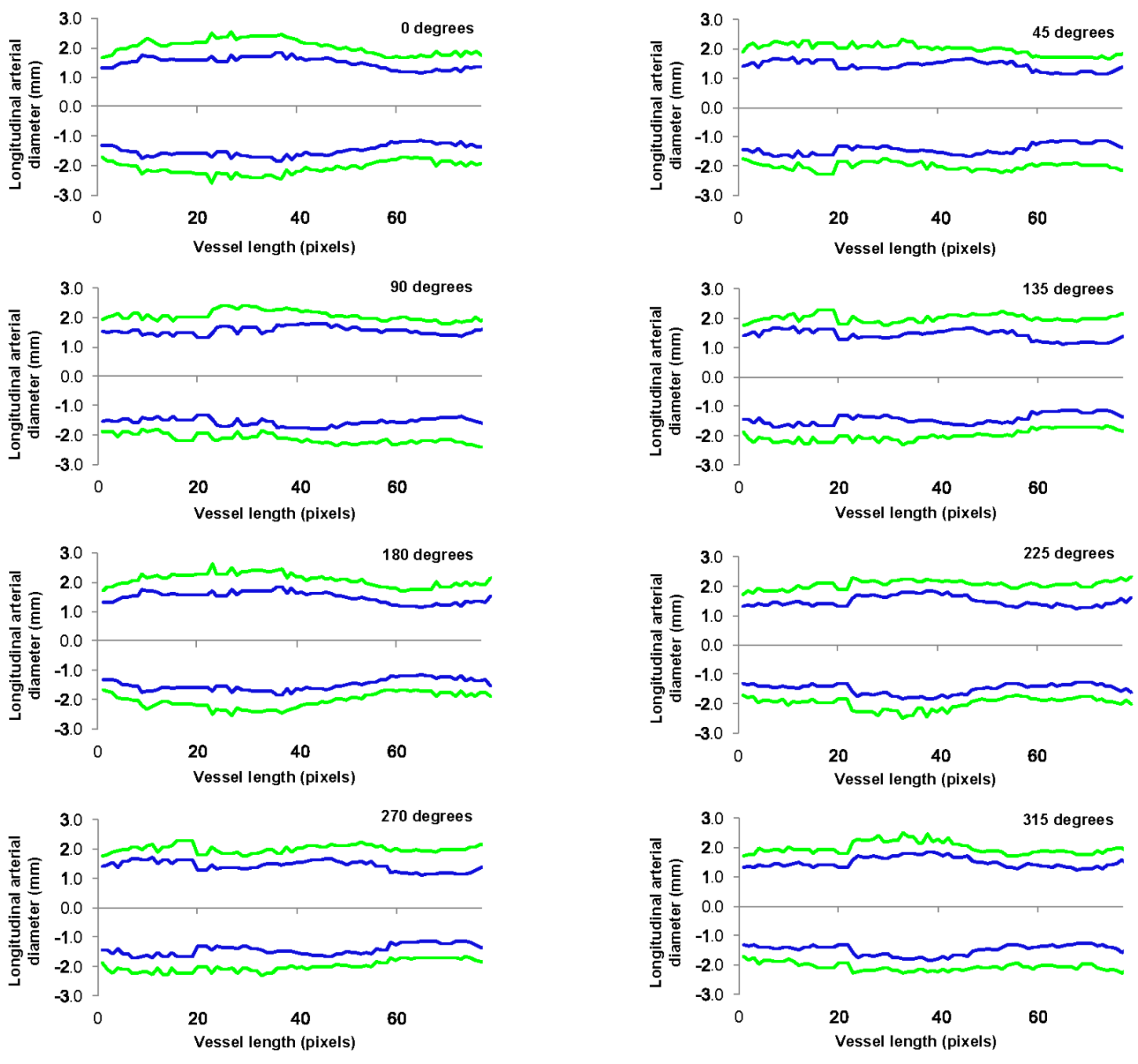

Supplementary Figure 4. Coronary silhouettes reconstructed by IVUS-images, by using row measurements. Each graph corresponds to measurements obtained in one of the investigated angles. The distance between the blue lines corresponds to lumen diameter, whereas the distance between blue and green lines corresponds to U-AWT and L-AWT.

Smoothing the IVUS diameter silhouettes by a sliding windows mathematical approach

Supplementary Figure 4 shows a large amount of noise in IVUS row measurements. Such noise is probably due to the longitudinal displacement of IVUS transducer and motion artifacts due to heart cycle, e.g. changes in coronary artery diameter and cyclic back-and-forth movements of the transducer in the longitudinal vessel direction associated to the cyclic change in the blood flow while the heart muscles are contracting and relaxing. As this noise may hinder the right interpretation of the measurement process, measurements of arterial lumen diameter (blue lines in Supplementary Figure 4) and measurements of U-AWT and L-AWT (distance between blue and green lines in Supplementary Figure 4) derived from IVUS cross-sectional row measurements were smoothed (Supplementary Figure 5). Smoothing was performed by using the same sliding window mathematical approach described for smoothing interfaces of QCA-images.

Specifically, two “sliding windows” averaging 5 consecutive frames, were moved along the vessel axis by consecutive steps of one frame. The first sliding window was moved from the first to the last frame of the IVUS pullback. The second was moved in the opposite direction (i.e. from the last to the first of the IVUS pullback). The values obtained at each step (i.e. the average of the 5-consecutive IVUS-frames) by the two sliding windows were then averaged and the average was plotted vs the length of the segment to obtain the final blue and green lines showed in Supplementary Figure 5. IVUS silhouettes were then compared with those obtained on original QCA-images (for validation of measurements on lumen diameter) and with those obtained on ACM-reprocessed QCA-images (for validation of measurements on both lumen diameter and arterial wall thickness).

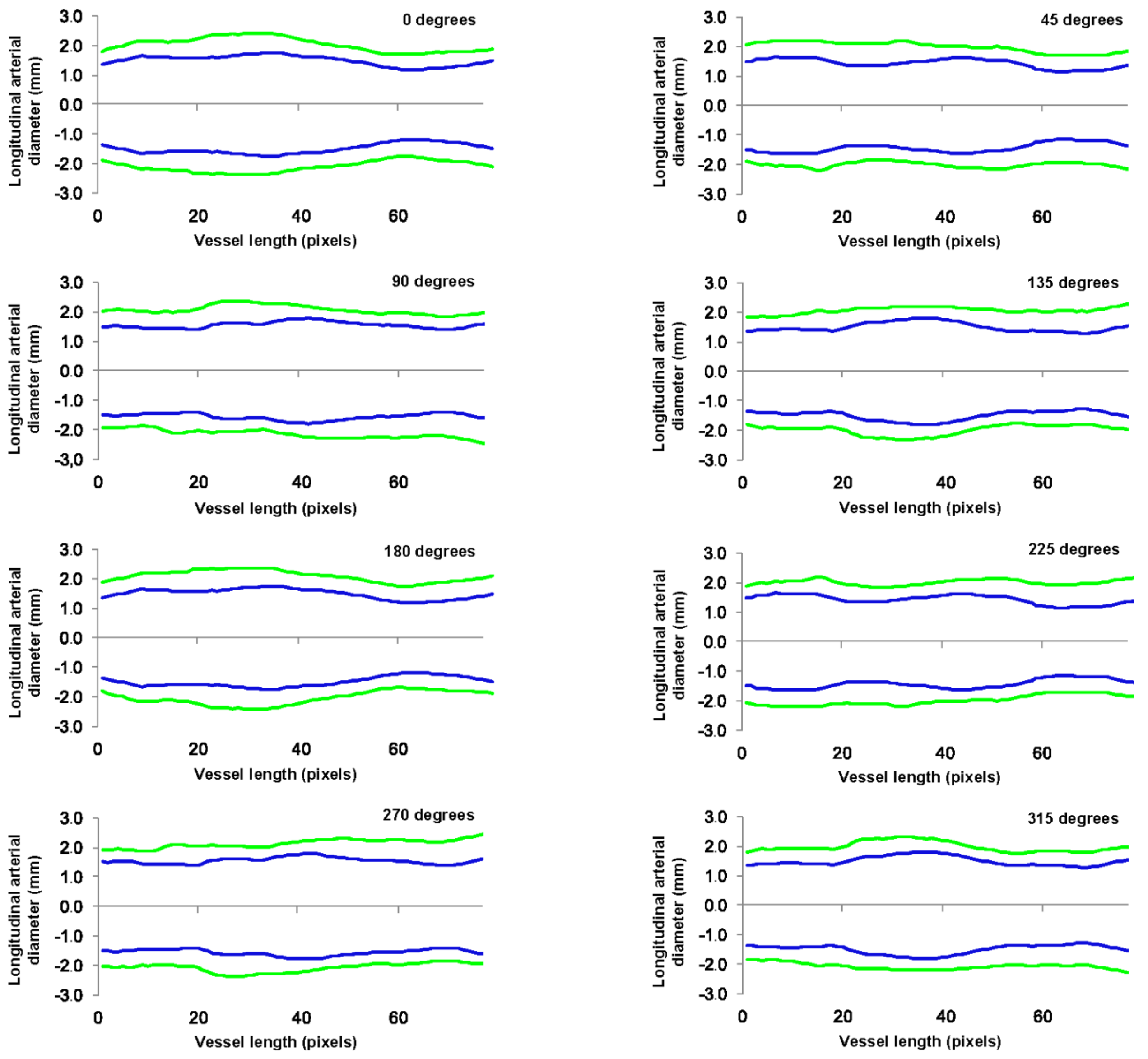

Supplementary Figure 5. Coronary silhouettes reconstructed by IVUS-images by using a sliding window mathematical approach. Each graph corresponds to measurements obtained in one of the investigated angles. The distance between the blue lines corresponds to lumen diameter, whereas the distance between blue and green lines corresponds to U-AWT and L-AWT.

Management of bias in the alignment procedure

Once obtained the longitudinal silhouettes and before considering which one of the silhouettes obtained in the eight IVUS angles could match with the single silhouette produced by QCA, it was necessary to face the problem of the longitudinal alignment. To minimize possible bias in the alignment procedure (i.e. to ensure that the IVUS and QCA ROIs considered for the validation study were exactly the same) QCA-images were used as roadmaps (Supplementary Figure 6).

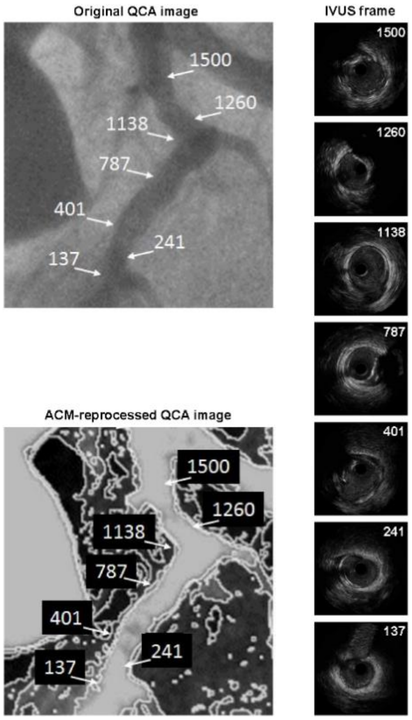

Supplementary Figure 6. Coronary angiogram showing the exact location of IVUS frames on the QCA-image. White numbers on the IVUS frame indicated the position on *left anterior descending* coronary artery. The IVUS-images ranged from 241 to 1500 and showed the presence of atheromatous lesions unrevealed by angiography. At distal reference point (image 137), the IVUS showed the absence of significant atheroma.

The path of IVUS transducer pulled back in the coronary segments as well as the start, middle and end fiduciary anatomical landmarks (e.g. aorto-ostial junction, branching vessels, bifurcations, etc), visible on both QCA and IVUS modalities, were accurately identified and meticulously matched. Anatomical landmarks allowed a macroscopic alignment only. For the microscopic alignment, the longitudinal match between QCA and IVUS segments was furtherly optimized by using a prepared-in-house software. Such software plotted on a graph the smoothed silhouettes of the lumen derived by both IVUS and original or ACM-reprocessed QCA-images. The software also allowed to progressively shifting the smoothed lumen lines by 10-IVUS frames increments until the concordance between IVUS-lumen and QCA-lumen silhouettes was maximized at visual comparison (8). Once that the longitudinal alignment was optimized, the software also allowed to automatically compare the lumen silhouette obtained by QCA with the silhouettes derived by the eight set of IVUS lumen contours obtained with 45-degree rotations. Specifically, the reconstructed IVUS lumen

silhouette obtained in each of the eight angles was back-projected on the QCA lumen and the cumulative point-by-point difference between QCA and IVUS lumen diameters was calculated. Such difference was used as metric to identify the projection with the best fit between the QCA and the IVUS lumens. In fact, the angle of IVUS interrogation, which produced the smallest cumulative differences between IVUS-derived and QCA-derived lumens contours, was identified as the best angle of investigation (Supplementary Figure 7; left panel). Only after having identified such best angle of investigation, the lines depicting the structures supposed to be arterial walls were added on the plot for arterial wall comparisons (Supplementary Figure 7; right panel).

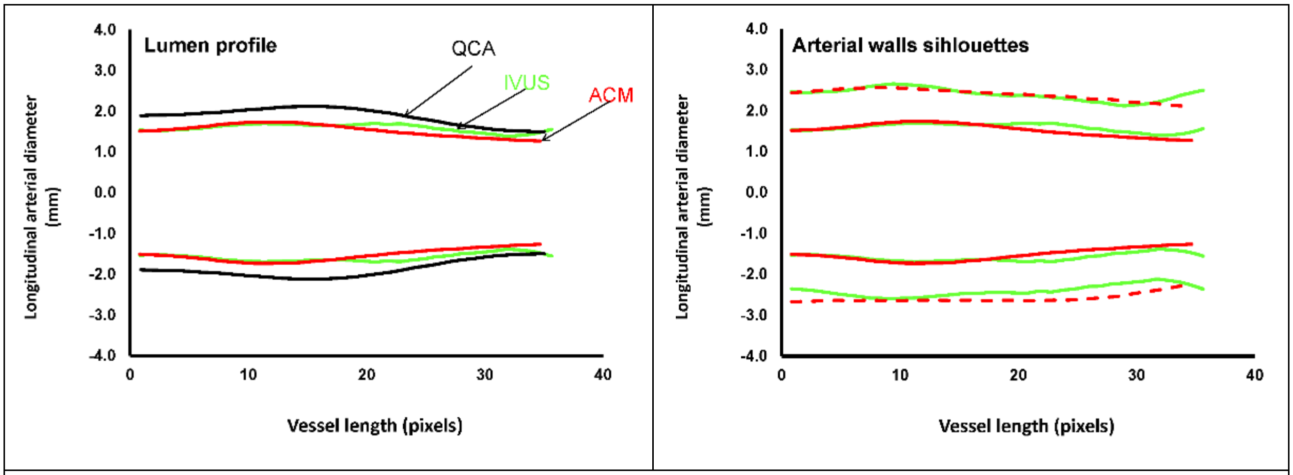

Supplementary Figure 7. Lumen and arterial wall silhouettes derived from original QCA-images, ACM-reprocessed QCA-images and from IVUS-images (gold standard). **Left panel**: Lumen silhouettes obtained by original QCA-images (black line), ACM-reprocessed QCA-images (red line) and IVUS images (gold standard). **Right panel**: the distance between the solid and dotted red lines denotes arterial wall silhouettes derived from ACM-reprocessed QCA-images, whereas the distance between the green lines denotes arterial wall silhouettes derived from IVUS cross-sectional measurements.

Appendix-3

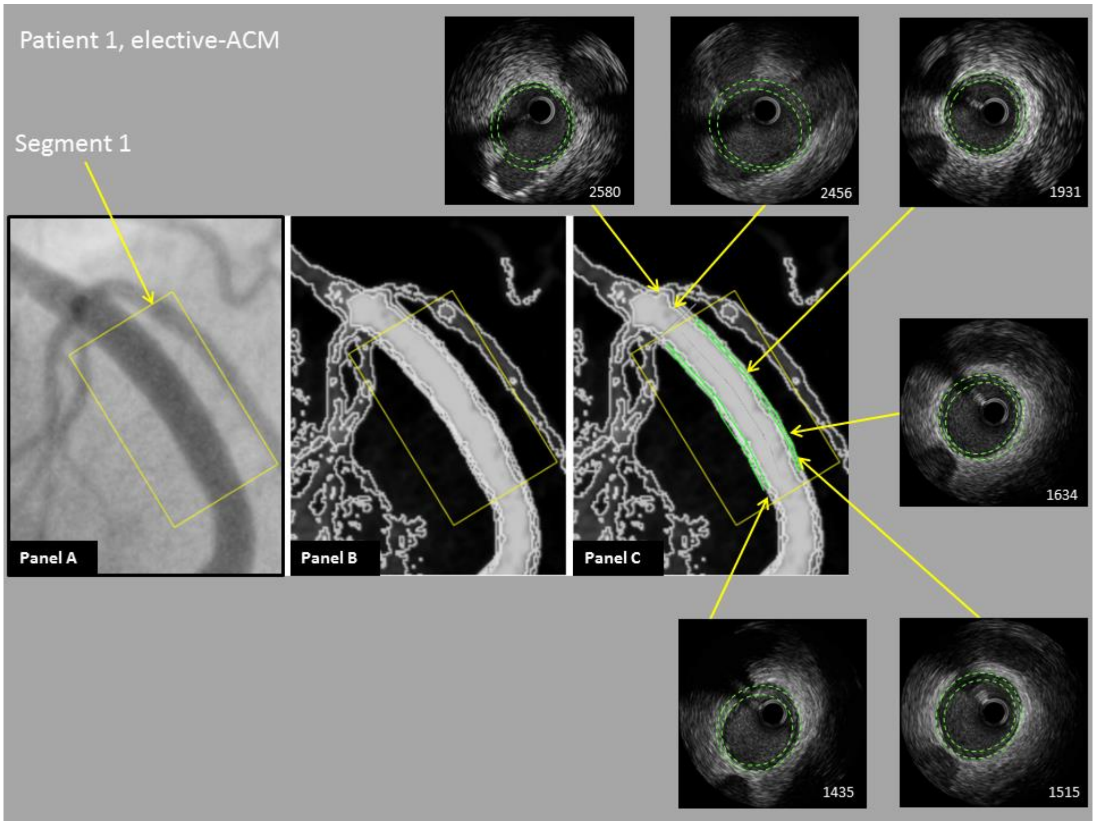

Supplementary Figure 8. Qualitative concordance of the shape of lumen and arterial wall silhouettes derived by ACM-reprocessed QCA-images and IVUS-images in patient 1, segment 1.

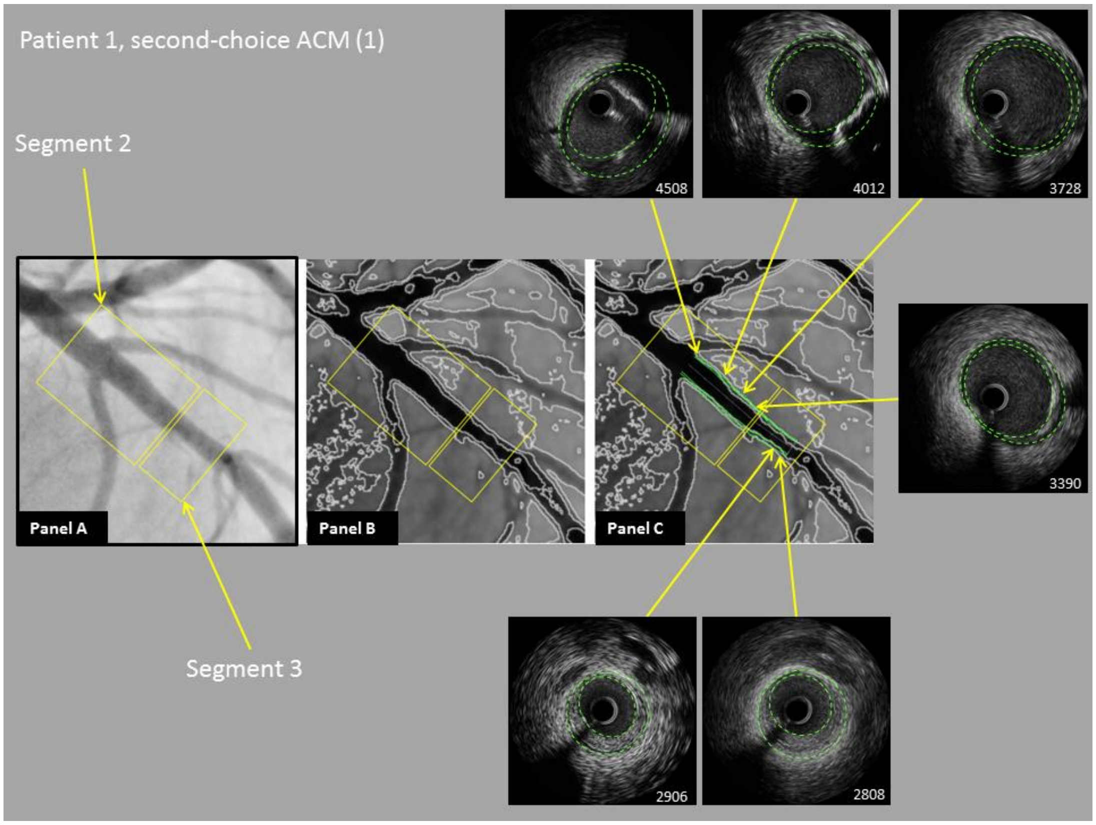

Supplementary Figure 9. Qualitative concordance of the shape of lumen and arterial wall silhouettes derived by ACM-reprocessed QCA-images and IVUS-images in patient 1, segments 2, 3.

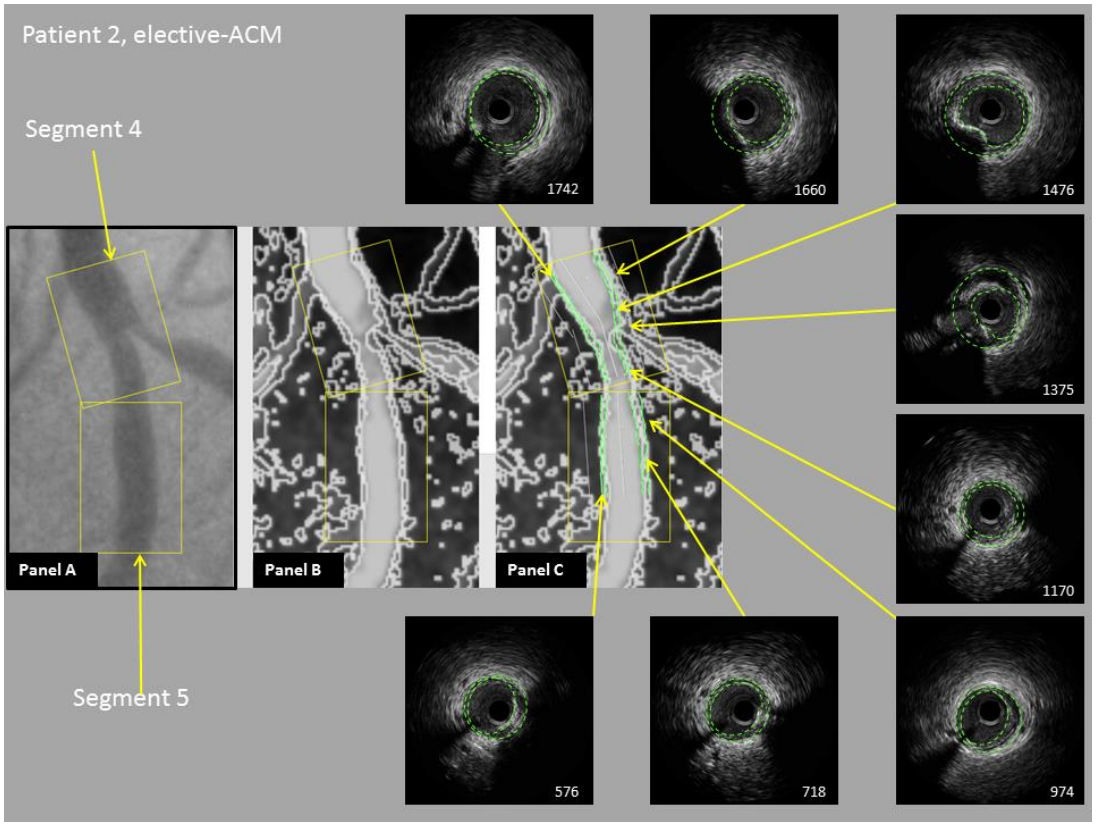

Supplementary Figure 10. Qualitative concordance of the shape of lumen and arterial wall silhouettes derived by ACM-reprocessed QCA-images and IVUS-images in patient 2, segments 4, 5.

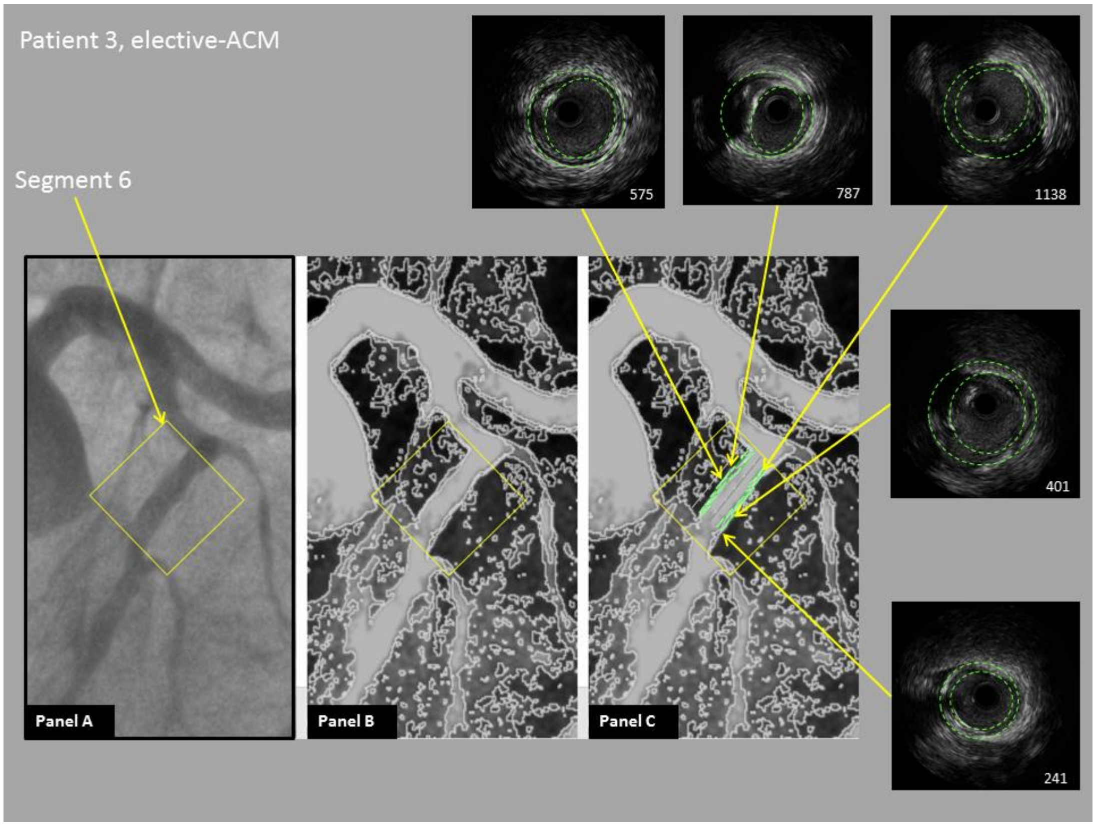

Supplementary Figure 11. Qualitative concordance of the shape of lumen and arterial wall silhouettes derived by ACM-reprocessed QCA-images and IVUS-images in patient 3, segment 6.

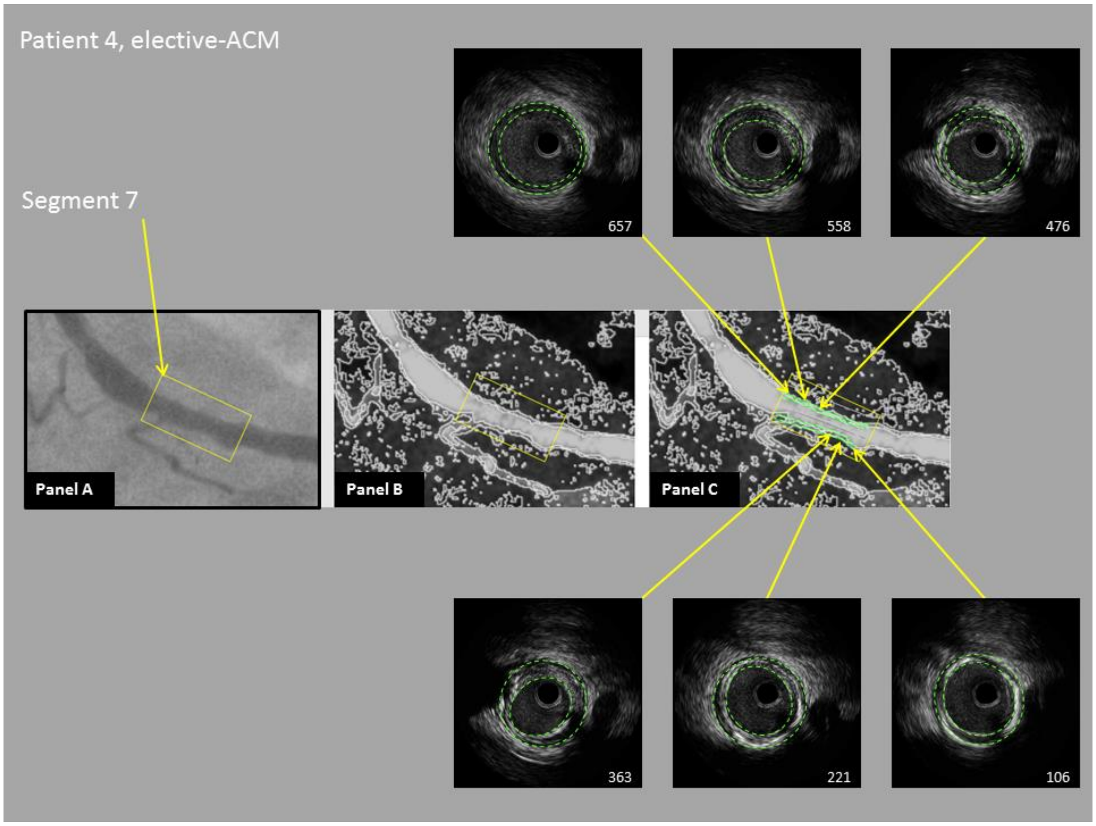

Supplementary Figure 12. Qualitative concordance of the shape of lumen and arterial wall silhouettes derived by ACM-reprocessed QCA-images and IVUS-images in patient 4, segment 7.

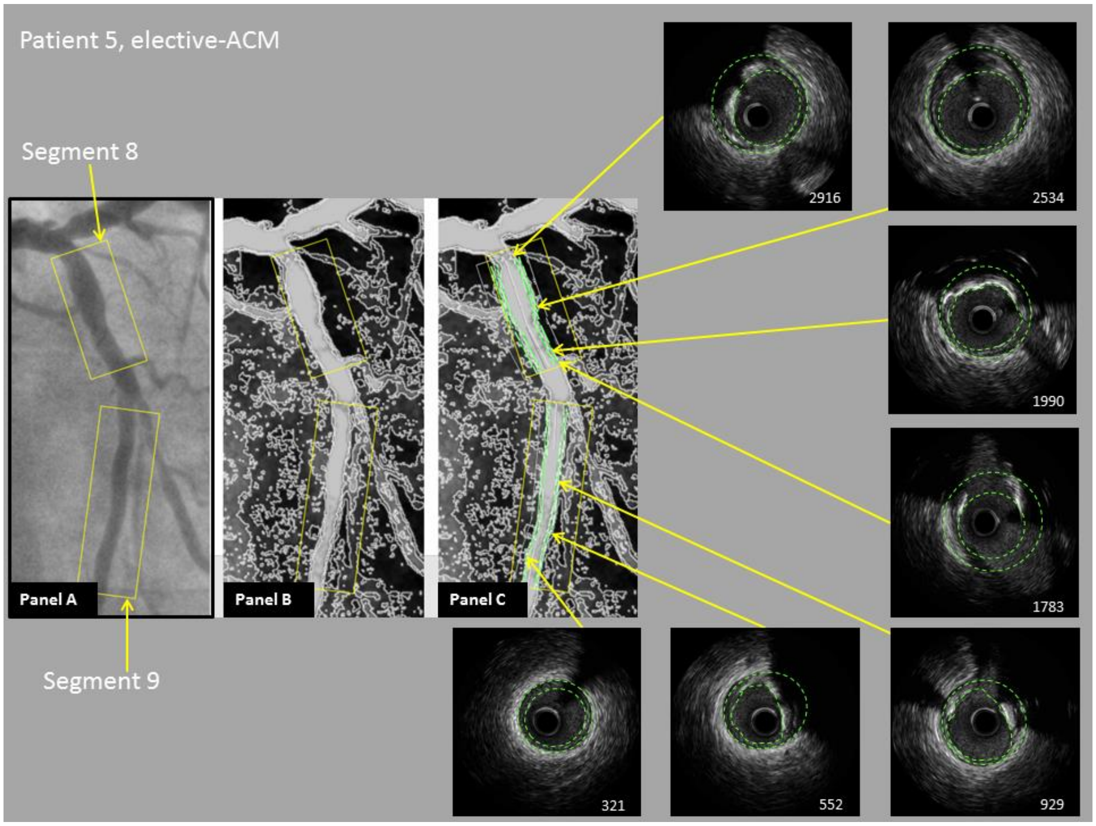

Supplementary Figure 13. Qualitative concordance of the shape of lumen and arterial wall silhouettes derived by ACM-reprocessed QCA-images and IVUS-images in patient 5, segments 8, 9.

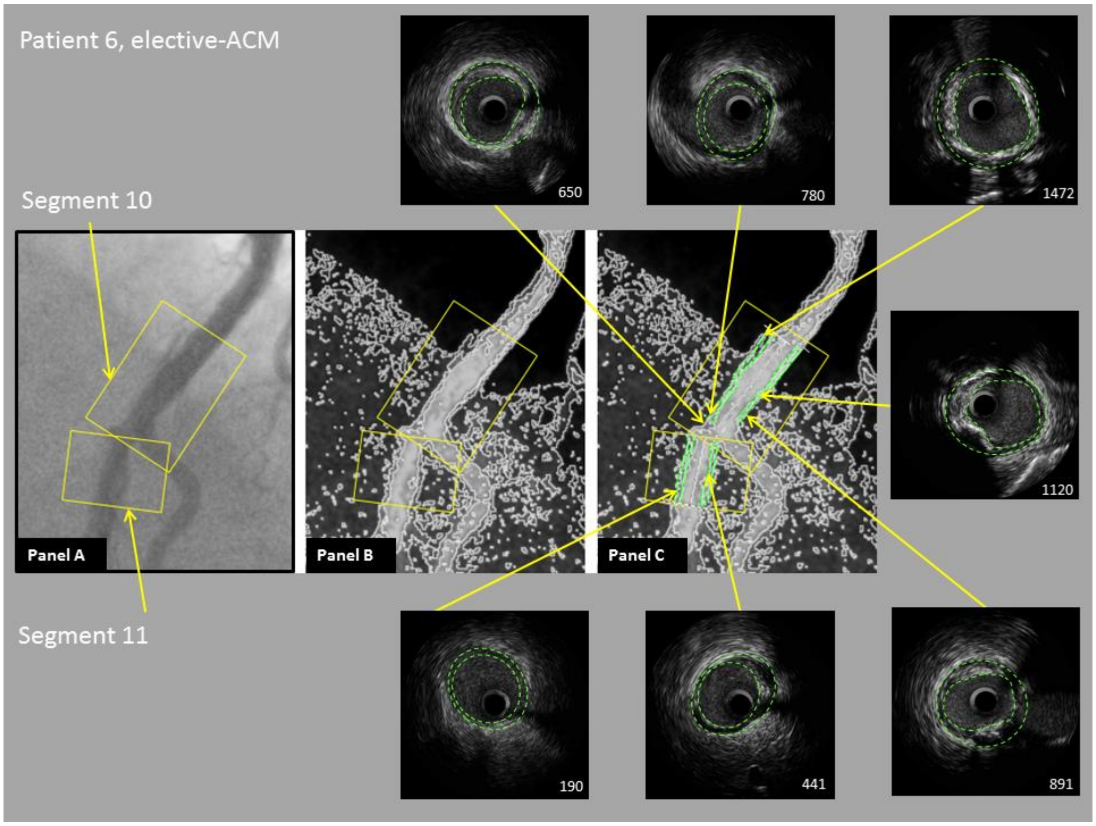

Supplementary Figure 14. Qualitative concordance of the shape of lumen and arterial wall silhouettes derived by ACM-reprocessed QCA-images and IVUS-images in patient 6, segments 10, 11.

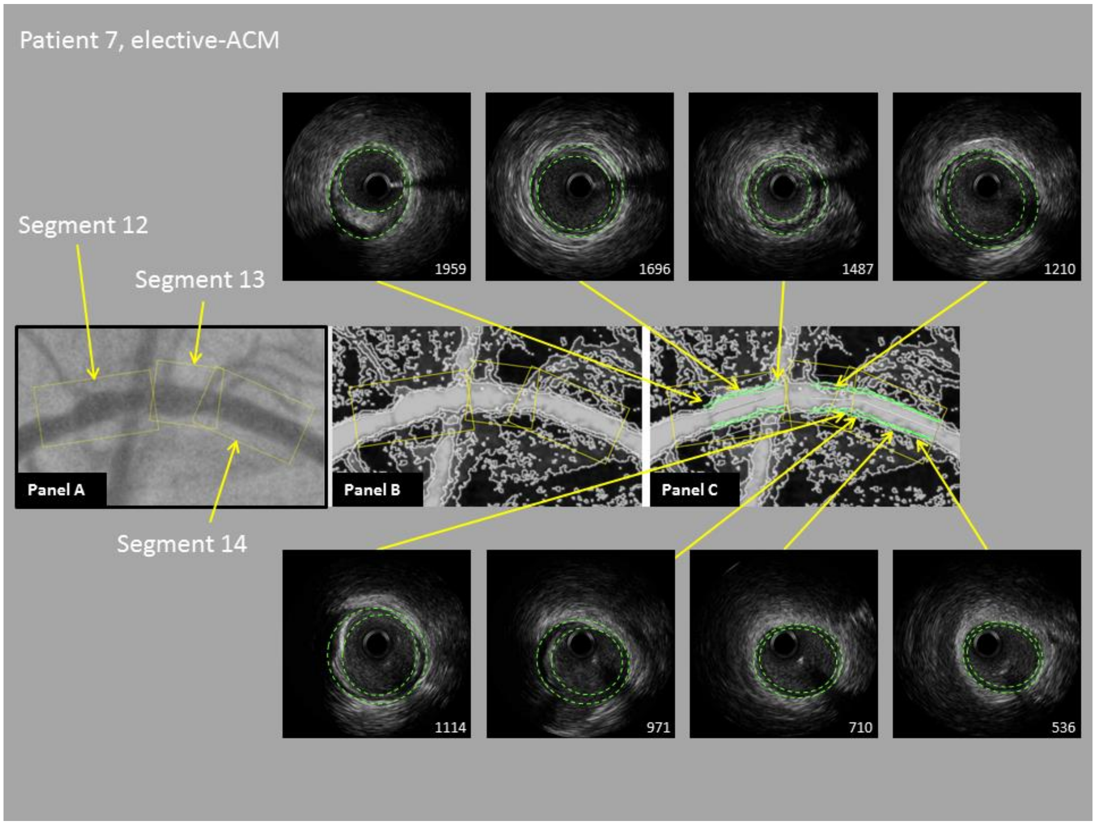

Supplementary Figure 15. Qualitative concordance of the shape of lumen and arterial wall silhouettes derived by ACM-reprocessed QCA-images and IVUS-images in patient 7, segments 12-14.

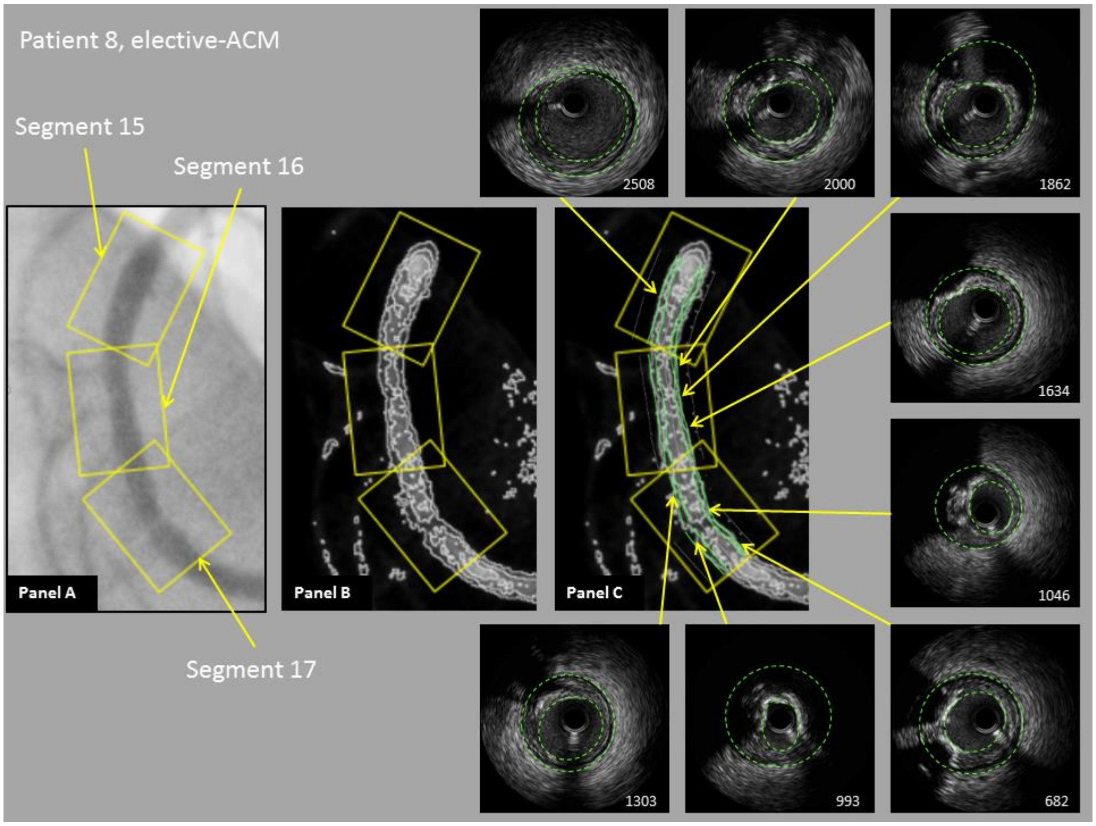

Supplementary Figure 16. Qualitative concordance of the shape of lumen and arterial wall silhouettes derived by ACM-reprocessed QCA-images and IVUS-images in patient 8, segments 15-17.

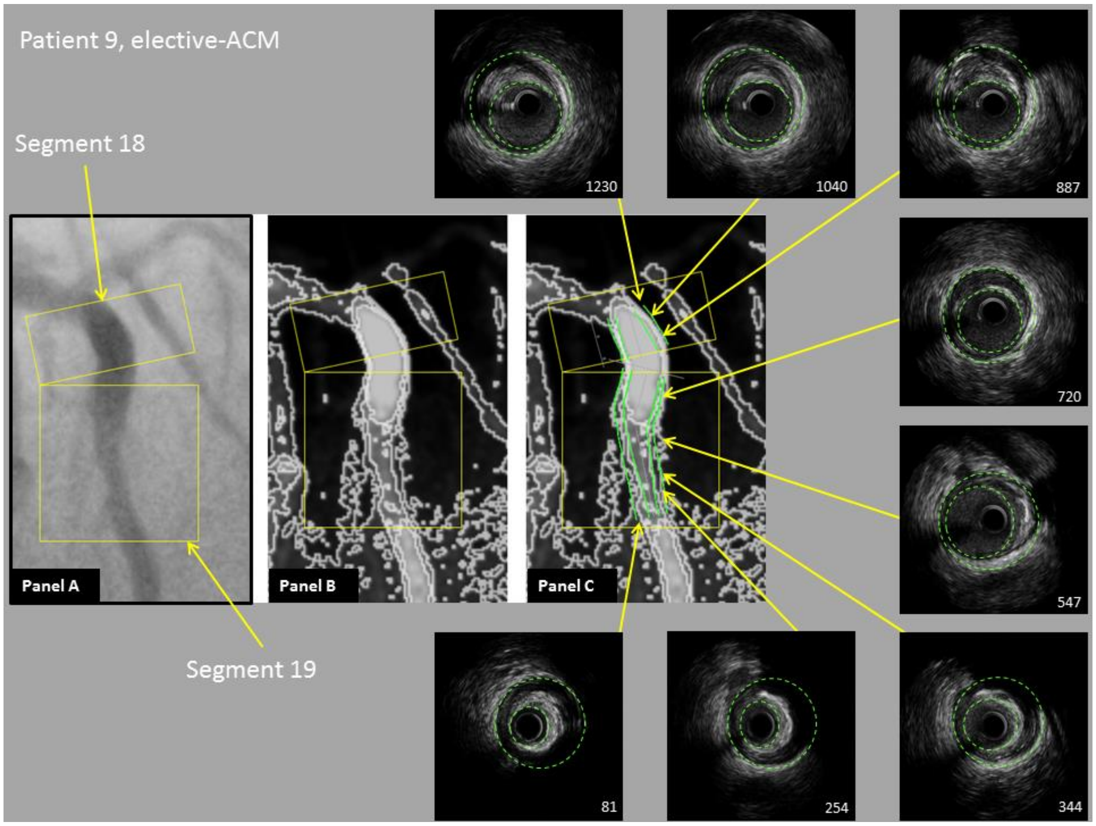

Supplementary Figure 17. Qualitative concordance of the shape of lumen and arterial wall silhouettes derived by ACM-reprocessed QCA-images and IVUS-images in patient 9, segments 18, 19.

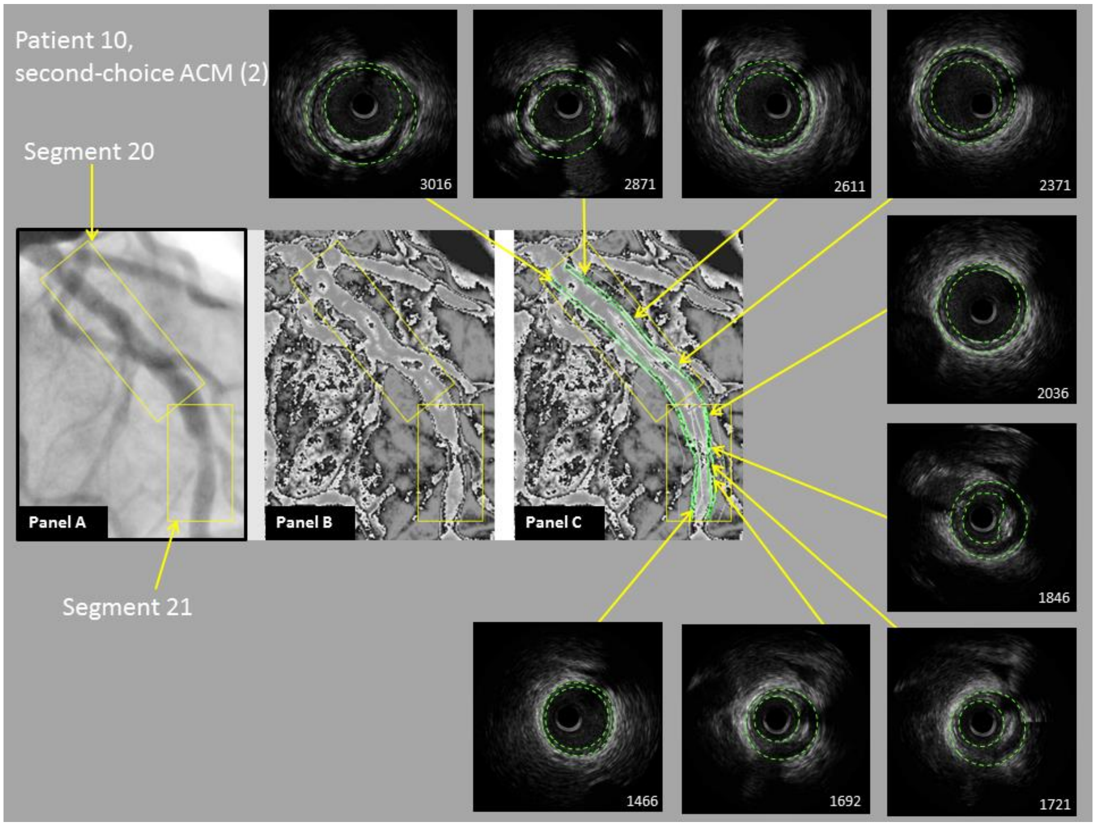

Supplementary Figure 18. Qualitative concordance of the shape of lumen and arterial wall silhouettes derived by ACM-reprocessed QCA-images and IVUS-images in patient 10, segments 20, 21.

SUPPLEMENTARY REFERENCES

1. Mancini GB, Abbott D, Kamimura C, Yeoh E. Validation of a new ultrasound method for the measurement of carotid artery intima medial thickness and plaque dimensions. *The Canadian journal of cardiology*. (2004) 20:1355-9.
2. Tu S, Holm NR, Koning G, Huang Z, Reiber JH. Fusion of 3D QCA and IVUS/OCT. *Int J Cardiovasc Imaging*. (2011) 27:197-207. doi: 10.1007/s10554-011-9809-2
3. Fuessl RT, Mintz GS, Pichard AD, Kent KM, Satler LF, Popma JJ, et al. In vivo validation of intravascular ultrasound length measurements using a motorized transducer pullback system. *Am J Cardiol*. (1996) 77:1115-8. doi: S0002914996001452 [pii]
4. Mintz GS, Nissen SE, Anderson WD, Bailey SR, Erbel R, Fitzgerald PJ, et al. American College of Cardiology Clinical Expert Consensus Document on Standards for Acquisition, Measurement and Reporting of Intravascular Ultrasound Studies (IVUS). A report of the American College of Cardiology Task Force on Clinical Expert Consensus Documents. *J Am Coll Cardiol*. (2001) 37:1478-92.
5. Touboul PJ, Prati P, Scarabin PY, Adrai V, Thibout E, Ducimetiere P. Use of monitoring software to improve the measurement of carotid wall thickness by B-mode imaging. *J Hypertens Suppl*. (1992) 10:S37-41.
6. Montorsi P, Galli S, Fabbiocchi F, Loaldi A, Trabattoni D, Grancini L, et al. Mechanism of cutting balloon angioplasty for in-stent restenosis: an intravascular ultrasound study. *Catheter Cardiovasc Interv*. (2002) 56:166-73.
7. Amato M, Montorsi P, Ravani A, Oldani E, Galli S, Ravagnani PM, et al. Carotid intima-media thickness by B-mode ultrasound as surrogate of coronary atherosclerosis: correlation with quantitative coronary angiography and coronary intravascular ultrasound findings. *Eur Heart J*. (2007) 28:2094-101. doi: ehm244 [pii]10.1093/eurheartj/ehm244
8. Gerretsen S, Kessels AG, Nelemans PJ, Dijkstra J, Reiber JH, van der Geest RJ, et al. Detection of coronary plaques using MR coronary vessel wall imaging: validation of findings with intravascular ultrasound. *Eur Radiol*. (2013) 23:115-24. doi: 10.1007/s00330-012-2576-1
